# Design and characterization of all 2D fragile topological bands

**DOI:** 10.1093/pnasnexus/pgaf285

**Published:** 2025-09-05

**Authors:** Samuel Bird, Chiara Devescovi, Pascal Engeler, Agnes Valenti, Doruk Efe Gökmen, Robin Worreby, Valerio Peri, Sebastian D Huber

**Affiliations:** Institute for Theoretical Physics, ETH Zurich, Zürich 8093, Switzerland; Institute for Theoretical Physics, ETH Zurich, Zürich 8093, Switzerland; Donostia International Physics Center, Paseo Manuel de Lardizabal 4, Donostia-San Sebastián 20018, Spain; Institute for Theoretical Physics, ETH Zurich, Zürich 8093, Switzerland; Institute for Theoretical Physics, ETH Zurich, Zürich 8093, Switzerland; Center for Computational Quantum Physics, Flatiron Institute, New York, NY 10010, USA; Institute for Theoretical Physics, ETH Zurich, Zürich 8093, Switzerland; James Franck Institute and Department of Statistics, University of Chicago, Chicago, IL 60637, USA; NSF-Simons National Institute for Theory and Mathematics in Biology, Chicago, IL 60611, USA; Institute for Theoretical Physics, ETH Zurich, Zürich 8093, Switzerland; Institute for Theoretical Physics, ETH Zurich, Zürich 8093, Switzerland; Department of Physics and Institute of Quantum Information and Matter, California Institute of Technology, Pasadena, CA 91125, USA; Institute for Theoretical Physics, ETH Zurich, Zürich 8093, Switzerland

## Abstract

Designing topological materials with specific topological indices is a complex inverse problem, traditionally tackled through manual, intuition-driven methods that are neither scalable nor efficient for exploring the vast space of possible material configurations. In this work, we develop an algorithm that leverages the covariance matrix adaptation evolution strategy to optimize the Fourier representation of the periodic functions shaping the designer material’s characteristics. This includes mass profiles or dielectric tensors for phononic and photonic crystals, respectively, as much as synthetic potentials applicable to ultra-cold atomic systems. We demonstrate our methodology with a detailed characterization of a class of topological bands known as “fragile topological,” showcasing the algorithm’s capability to address both topological characteristics and spectral quality, and demonstrating the experimental feasibility of realizing all of the classified fragile topological phases. This automation not only streamlines the design process but also significantly expands the potential for identifying and constructing high quality designer materials across the wide range of platforms, and is readily extendable to other setups, including higher-dimensional and nonlinear systems.

Significance Statement The design of metamaterials with specific physical properties is a difficult challenge as an inverse problem on a generically large and nonsmooth design space, and is typically done by hand at great time expense. We present a solution to this problem through our method, demonstrated on the high-throughput design of metamaterials that possess any and every type of fragile topology. As well as targeting topological properties, we demonstrate our method’s ability to efficiently optimize spectral qualities of the metamaterial, aiding experimental access. Alongside methodological developments, this work makes it clear that, while the fragile topological phases had been classified previously, it should in fact be feasible to design all of them and experimentally realize them in a metamaterial.

## Introduction

Since the advent of topology in condensed matter physics with the theoretical explanation of the Integer quantum Hall effect (1–4), it has been used repeatedly, e.g. to construct field theories to explain the interacting version of the quantum Hall effects (5–9) or to categorize the electronic phases in all crystalline materials (10, 11). In the last example, topology led to predicted observations rather than explaining them in hindsight. With the recent advances in *designer materials*, a new application of topology in physics emerged. In photonic (12) and phononic materials (13, 14), nano-structured electronic systems (15, 16), as well as in ensembles of cold atoms (17–21), custom periodic structures are created to induce artificial Bloch bands with desired properties. The properties of these Bloch bands are then used to either achieve a sought-after functionality of the architectured material, e.g. a topological laser (22), or to observe new physical phenomena (23–26).

Encoding a functionality or a new physical phenomenon in terms of a topological index is a powerful tool for two reasons. First, such an index serves as a simple and stable objective function in the *design* of a periodic structure, may it be a specific optical lattice for cold atoms, an arrangement of gates defining a periodic potential for electrons in a 2D electron gas, or an architectured photonic or phononic crystal. Second, the achieved functionality has the chance to *display some protection* against fabrication or implementation imperfections.

Unfortunately, finding a periodic structure whose bands have a prescribed topological index is a complicated inverse problem with no generic solution. Despite this complication, the field of designer topological materials enjoyed remarkable success over the years (15, 25, 26, 28–33). However, in all the above examples a periodic structure had to be conceived and optimized by hand, typically guided by a simple discrete model. This approach is unsuitable for large-scale, high-throughput explorations, or for problems where either no simple discrete model is known or fabrication constraints severely obstruct the intuition-based workflow. Here, we describe a systematic approach to this challenge and employ our methodology on one specific example, presenting an exhaustive characterization of a class of topological bands known as “fragile topological” in *all* the aforementioned platforms, an impossible endeavor without a fully automated algorithm.

How are such topological bands typically found? For microscopic quantum materials, databases such as the Inorganic Crystal Structure Database (34) serve as a shopping list to identify new topological systems (11). Here, we focus on the intermediate to large-scale designer materials described above. The Bloch bands in all of these systems are described by a partial differential equation where one of their coefficients $f(\vec{r}\;)$ is a periodic function of space. This can be a periodic potential $V(\vec{r}\;)$ for the Schrödinger equation of ultra-cold atomic systems (Supplementary material), a dielectric function $\epsilon (\vec{r}\;)$ in the Maxwell equations for photonic crystals (Supplementary material), or a mass distribution $\sigma (\vec{r}\;)$ for the Poisson equation describing the vibrations in thin membranes (Supplementary material).

Designing intermediate to large-scale materials to showcase sought-after (topological) properties comes with advantages but also challenges compared to their microscopic counterparts. We are not constrained by combinations of atoms that form chemically stable compounds like in Ref. (11), but our design space is given by a set of reasonably well-behaved functions of the spatial coordinates. However, this design flexibility comes at a steep algorithmic price: Optimizing a structure $f(\vec{r}\;)$ with the goal to achieve some topological nature of the elementary excitations is suffering from all the challenges inherent to high-dimensional optimization routines. Here, we overcome this challenge using a modern evolution strategy in the form of the Covariance Matrix Adaptation Evolution Strategy (CMA-ES) (35, 36).

We base our approach on topological phases that can be detected by eigenvalues of crystalline symmetries. There are a number of reasons for this choice. First, almost all known topological insulators have a crystalline counterpart, including Chern insulators (37) or $Z_{2}$ (38) insulators, to name just two examples. Second, for many designer materials, crystalline symmetries are a natural choice, as one typically replicates a local pattern periodically, with the local pattern obeying the symmetries of a point group e.g. rotations, mirrors, or glide reflections.

Finally, eigenvalues of crystalline symmetries are straightforward to compute and hence serve as a computationally cheap input for a large scale search of topological bands.

**Fig. 1.**
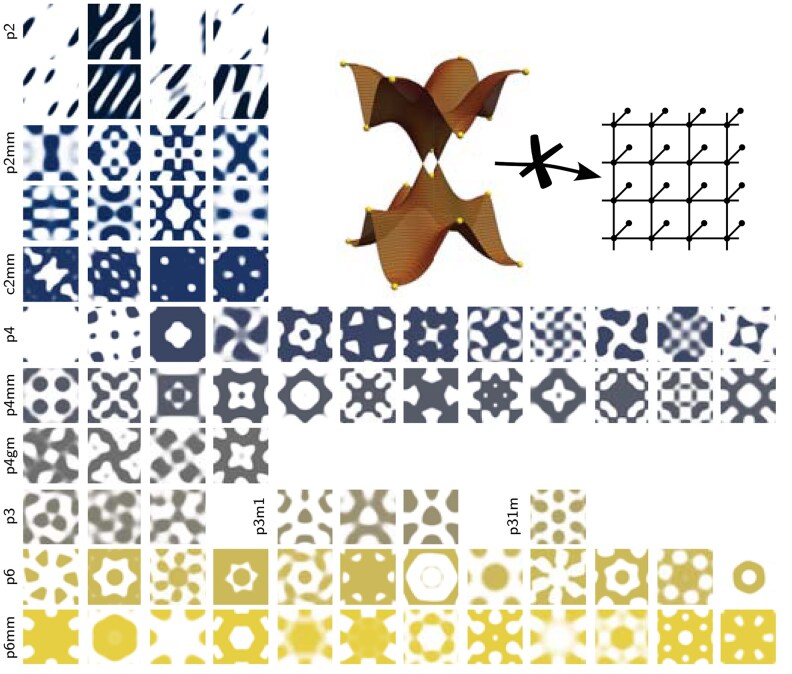
Complete catalog of samples with fragile bands. Inset: Illustration of a topological set of bands that cannot be written in terms of a tight-binding model. The yellow dots indicate the high-symmetry points in the Brillouin zone where the eigenvalues of the space group symmetries determine the topology. Main panel: For each wallpaper group we present the unit cell of a 2D structure which describes a mass profile σ(x,y) or a periodic dielectric constant ϵ(x,y) that leads to fragile topological bands for the phonons or photons, respectively. We find structures for all the 79 distinct phases that have been theoretically predicted in Ref. (27).

Concretely, we use the framework of topological quantum chemistry (38, 39): Sets of isolated bands are deemed topological if one cannot write them in terms of a basis of exponentially localized symmetric orbitals, see Fig. 1. It turns out that there are two distinct ways to fail this test: Either bands are topological and can only be trivialized by adding another set of topological bands. All well-established topological insulators having a Chern number or a $Z_{2}$ index fall into this category of “stable topology.” However, there arises the possibility that bands are topological but can be trivialized by a band that is itself trivial. This constitutes the class of “fragile topology.” It turns out by simple inspection (40), that in two dimensions without spin-orbit interaction, which for the chosen designer-materials platforms is the standard rather than the exception, fragile topology is the only possibility. Moreover, we will constrain ourselves to systems that do not break time-reversal symmetry for simplicity. What is the significance of these fragile states? Fragility has a large number of physical consequences, such as in disordered systems (41), flat-band superconductors (42, 43), or in how the spectrum reacts to defects (26), and with fragility known to produce fractional corner charges through the filling anomaly (44). Acoustic systems offer a natural route to exploring this physics, since the filling anomaly underlying its corner charges can be measured through gauge-flux experiments (45). It is also the basis of recent realizations of Bosonic topological insulators with helical edge states (26, 30).

All 2D eigenvalue-indicated fragile phases have been tabulated according to their symmetry eigenvalues in Ref. (27). Each phase can be labeled by the irreducible representations (irreps) of the wallpaper groups at high symmetry points in reciprocal space. For example, for the wallpaper group p4mm with a four-fold rotation axis and two mirror planes, the possible irreps are $\Omega_{1},\ldots ,\Omega_{5}$ for Ω=Γ=(0,0) and Ω=M=(1/2,1/2), respectively, as well as $X_{1},X_{2}$ for the point X=(1/2,0). The points in the Brillouin zone are written with respect to the reciprocal lattice vectors. An example of a set of connected fragile bands in the table of Ref. (27) is


(1) $$\Gamma_{1}\Gamma_{4},M_{5},X_{1}X_{2};$$

where at the points *Γ* and *X* two singly degenerate bands are realized which are joined in the 2D irrep $M_{5}$ at the *M* point (Supplementary material). There are 79 different distinct sets of irreps, or roots, which describe fragile topological bands in 11 of the 17 wallpaper groups (46). While some of these have been experimentally observed (26), it is not known if all of them can occur in a realistic material. To find concrete material examples for all of them, to characterize those structures, and optimize them for scientific investigations or technological applications is the concrete challenge we want to meet with our proposed design algorithm.

## Methods

Let us outline the algorithm. Our design space is given by 2D periodic functions $f(\vec{r}\;)$. We parameterize these functions via their Fourier-coefficients $f(\vec{r}\;)=\sum_{n}u_{n}e^{i{\vec{k}}_{n}\cdot \vec{r}}$. The ${\vec{k}}_{n}$ are chosen to reflect the specific lattice in a given wallpaper group and the expansion coefficients $\left\{u_{n}\right\}$ fulfill the necessary relations for different *n*’s, such that all symmetries of the wallpaper group are realized (46, 47). For some platforms we also discretize the profile by using $\tilde{f}(\vec{r}\;)=f_{0}+\frac{\;f_{1}-f_{0}}{2}\{1+\text{tanh}[f(\vec{r}\;)/\xi ]\}$ with ξ→0 to encode a system where $\tilde{f}$ only takes the two values $f_{0}$ and $f_{1}$. This is relevant for Photonic crystals where it is hard to produce a generic continuous dielectric function. The set of independent $\left\{u_{n}\right\}$ make up the high-dimensional continuous search space for our algorithm.

We start the algorithm with a random set of $\left\{u_{n}\right\}$ and solve the partial differential equation (PDE) at the high-symmetry points in the Brillouin zone. We do this using a finite element method (FEM). We design our own FEM meshes to ensure efficient enforcement of crystalline symmetries, which we then pass to the FEM package FEniCS, which generates the relevant FEM problem for each of the systems of interest (48–50). This formulates the PDE as a sparse matrix eigenvalue problem, that we diagonalize ourselves using LAPACK (51). For each high symmetry point, the solutions form irreps of the respective little group, which can be labeled by the standard symbol of the high-symmetry point and a numerical label (Supplementary material). When ordering the symbols of a given solution by eigenvalues, these form words of the form (again on the example of p4mm)


(2) $$\Gamma_{1}\Gamma_{1}\Gamma_{4}\Gamma_{4}\ldots ,M_{1}M_{1}M_{5}M_{4}\ldots ,X_{1}X_{2}X_{2}X_{1}\ldots .$$

In a first step, one can *bundle these bands* into set of bands where symmetry compatibility relations require them to be *connected*


(3) $$\Gamma_{1}^{a}\Gamma_{1}^{b}\Gamma_{2}^{b}\Gamma_{4}^{c}\ldots ,M_{1}^{a}M_{5}^{b}M_{4}^{c}\ldots ,X_{1}^{a}X_{2}^{b}X_{2}^{b}X_{1}^{c}\ldots ,$$

where those irreps with the same superscript belong to a given bundle. In this way, we confirm the likely presence of an energy gap above and below the bundle, which we confirm along the high symmetry lines in post-processing. At this step, we aim only for an energy gap everywhere, that is, the target bundle is energetically separated from neighboring bands. Later, our spectral optimization using CMA-ES refines this separation into a complete gap along the high symmetry lines, establishing a full forbidden energy window.

In the next step, we can check if any of the bundles conform with the sought-after roots of Ref. (27). If this is the case [in the example above the bundle labeled *b* realizes the root of Eq. 1], one can store $\left\{u_{n}\right\}$ for later optimization. If none of the bundles correspond to the target, one move on to the next random set $\left\{u_{n}\right\}$. In CMA-ES, the parameters $\left\{u_{n}\right\}$ are drawn from a normal distribution $N(\{{\bar{u}}_{n}\};C_{nm})$, characterized by the means ${\bar{u}}_{n}$ and the covariance matrices $C_{nm}=\bar{\left(u_{n}-{\bar{u}}_{n})(u_{m}-{\bar{u}}_{m}\right)}$. The coefficients ${\bar{u}}_{n}$ and $C_{nm}$ are then adapted in an evolution strategy to optimize a given cost function (35, 36).

Finally, when a topological band is found, one can optimize further properties by using a refined cost function. A typical example would be to push for the maximum possible band gap without destroying the topological character. Such mixed-cost functions are straightforward to implement, and we will come back to this point below.

## Results

We now present the results achieved with this algorithm searching for all possible fragile bands. For each of the 79 roots, we display the corresponding structure in Fig. 1. Shown are structures $f(\vec{r}\;)$ that lead to topological bands for electromagnetic TM and TE modes or phonons in thin membranes. Examples for the other platforms and the associated Bloch bands are shown in Supplementary material. These structures represent the main result of this work where we establish that all fragile bands can in principle be realized.

**Fig. 2.**
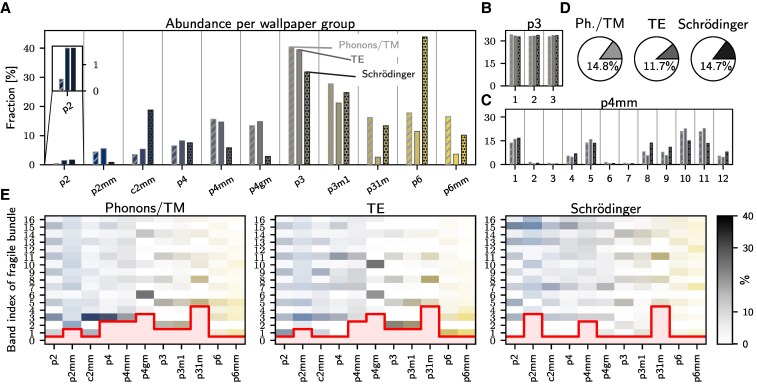
Statistical analysis of fragile topological bands. a) For each wallpaper group we show the likelihood that a random structure harbors a fragile topological band amongst its lowest 15 bands. The abundance of these topological bands are shown for phonons, photons, and the Schrödinger equation in a periodic potential. b) For the group p3, all three distinct fragile bands are equally likely to occur, independent of the platform. c) For p4mm, there are strong variations of the relative abundance. d) Averaged over all wallpaper groups the three studied platforms show a similar likelihood that a random structure harbors a fragile topological bands bundle. e) For each of the studied platform, we display how likely it is for a topological bundle to occur in the *n*’th band. The darker shade of the respective color indicates a higher likelihood. In the bands below the red line, no topological bundles were found. One observes that for some groups, e.g. in p31m we cannot find any topological bundle among the five lowest bands.

To highlight the power of the algorithm, we move to a detailed statistical analysis of structures containing fragile roots. The most pressing questions one may have are: how likely is it to find a topological band? Does it depend on the wallpaper group? Are there classes of fragile bands that are harder to realize than others? And finally, can one target the lowest Bloch bands only, or does one have to consider highly excited bands? In Fig. 2, we answer all the above questions.

In each wallpaper group, we draw random structures $\left\{u_{n}\right\}$ until we obtain 10,000 samples with at least one fragile root among the 15 lowest bands. For each of the wallpaper groups and all the three studied platforms, we then report what fraction are topological in Fig. 2(a). We observe that this depends strongly on the wallpaper group, and to a lesser extent, on the studied platform. One important observation is that for the group p3 for all platforms and for p6 for the Schrödinger equation, almost half of the random samples contain a fragile root. Hence, these groups may serve as an excellent starting point if one wants to optimize further properties beyond the presence of topological bands.

Next, in Fig. 2(b) and (c), we showcase for the two groups p3 and p4mm how the relative abundance is distributed among the different roots. While for p3 all roots are equally likely, in p4mm there is a significant variation between the different roots. This difference between p3 and p4mm can be understood by the different structures of the specific roots (Supplementary material). Note, that there are also extreme outliers. For example, root #3 in the group p4gm is extremely hard to find. Only one out of $10^{5}$ random structures turn out to realize this root. We finally observe that overall fragile bands are more or less equally likely to occur in all the studied platforms (Fig. 2(d)).

It is difficult to find fragile bands in the lowest bundle. In Fig. 2(e), we show the relative abundance of fragile roots as a function of the band index of the fragile bundle per wallpaper group and platform. One can clearly observe that for some groups one needs to go to rather highly excited bands. This is mostly implied by the relatively complex irreps involved (the counterpart of *d*- vs. *s*-wave orbitals), in particular the nature of the mechanism that binds the bands into the fragile 2-bundle: bundles that are bound by 2D or conjugate irreps are more common, whereas those bound by the fact that each individual band is necessarily a Chern band (and hence barred from isolation by time reversal symmetry) are less common. The bundling mechanism of each root is outlined in Supplementary material.

**Fig. 3.**
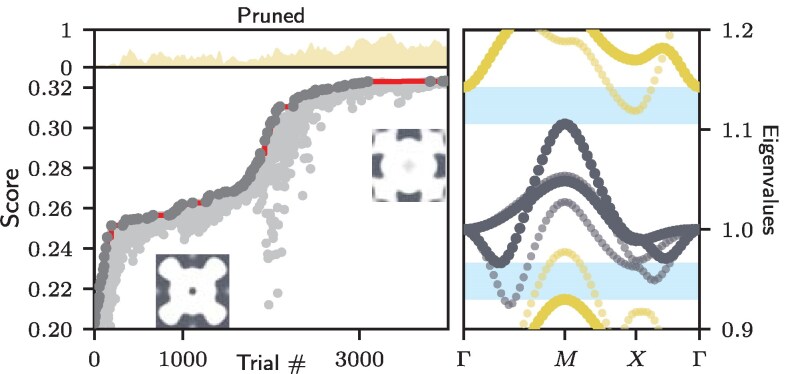
Gap optimization of a p4mm sample. Left panel: The score 1/C indicates the size of the gap to the adjacent bands as a function of the number of optimization steps. The insets show the initial and the final real-space structure. Also shown is the fraction of samples that are pruned as they lost the initial topological bands. Right panel: Bloch bands along the high symmetry lines of p4mm. The small gray dots show the bands of the initial structure where the topological bands largely overlap with the bands below shown in yellow. The big dots represent the final, optimized spectrum with a full band gap to the bands above and below indicated by the blue areas.

In the presented statistical analysis we did not focus on the size of the band gaps separating the topological from its adjacent bands. This is, however, for most practical applications one of the most important quality measures. We show on the example of a specific root in group p4mm how CMA-ES with an appropriate cost function can address this issue. Once the sought-after topological band is found in the form of a set of Fourier coefficients $\left\{u_{n}^{0}\right\}$, one can further optimize these coefficients for a large band gap. The evolution strategy of CMA-ES is optimally suited for this task: One generates additional samples by drawing new ones from $N(\{u_{n}^{0}\};\lambda_{n}\delta_{nm})$, with $\lambda_{n}\ll u_{n}^{0}$. The cost function for the update strategy of ${\bar{u}}_{n}$ and $C_{nm}$ can be chosen to take the form


(4) $$C=\frac{1}{\sum_{{\vec{k}}_{i}}\left(\epsilon_{n}({\vec{k}}_{i})-\epsilon_{n-1}({\vec{k}}_{i})+\epsilon_{m+1}({\vec{k}}_{i})-\epsilon_{m}({\vec{k}}_{i})\right)},$$

where *n* (*m*) labels the eigenvalues in the lowest (highest) band in the bundle and the ${\vec{k}}_{i}$’s are chosen from a suitable set of reciprocal vectors. In order not to spoil the topology of the bundle in question, we can simply prune those structures $\left\{u_{n}^{\alpha }\right\}$ which do not conform with the root one optimizes from the population that is used in CMA-ES. In Fig. 3, we show how a topological bundle that is initially overlapping with its neighboring band below, can be brought into a fully gapped system with a sizeable gap of more than five percent. The left panel of Fig. 3 shows the optimization curve and the right panel compares the initial and final energy gap.

## Discussion

Two observations are worth pointing out. First, the fraction of pruned samples is growing at later stages of the optimization: At this point, improving the gap at all seems to be only possible by introducing unwanted band-inversion. Second, the initial real space structure $f_{0}(\vec{r}\;)$ and the final $f_{\mathrm{final}}(\vec{r}\;)$ have very little in common with each other. This fact underlines why optimizations by hand or those parameterized by simple geometric motifs often do not yield satisfactory results.

**Fig. 4.**
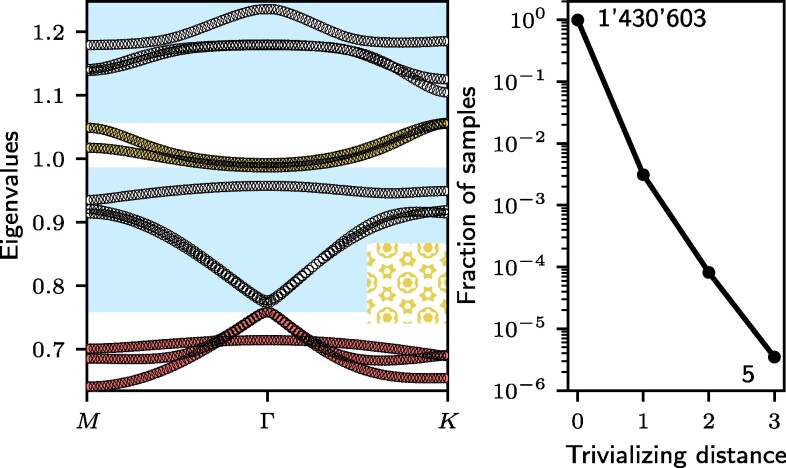
Trivializing distance. Left panel: Dispersion relation of a sample (shown in the inset) hosting a topological bundle (yellow) where the trivializing bundle (red) is separated by two “spectator” bundles shown in white. For the purpose of topology, the effective gaps shown in blue are relevant. Right panel: Relative likelihood for a fragile topological bundle to be separated by 1, 2, or 3 bundles from its trivializing one. The graph is based on more than 1.4 million samples, however, the overwhelming majority has its trivializing band direct adjacent.

Another way to protect the topology is by separating the fragile bands as much as possible from the trivializing ones. Remember that fragile bundles can be trivialized by trivial bands. However, not any trivial band can do that job. By post-selecting those structures where the trivializing band is far away from the bundle in question, one can achieve the same goal as above without the need to optimize any gap. In Fig. 4(left panel), we show one example where the fragile bundle is surrounded by bundles that do not trivialize it, essentially inducing a protecting gap to the one that does trivialize the bundle. One may ask how likely such a situation is. In Fig. 4(right panel), we show the number of samples as a function of the distance to their trivializing band. While one can see that this distance is exponentially suppressed, we do find distances of up to three bundles. To be precise, the “trivializing distance” is the number of bands that lies between the target fragile bundle and the nearest band bundle that possesses the EBRs which, when combined with the EBRs of the target bundle, would be trivial (decomposable as a sum of band representations). So, a trivializing distance of 0 would mean that there is a band bundle adjacent to the target bundle which possesses the trivializing EBR.

## Conclusion

In summary, we presented an algorithm to find topological bands by using the covariance matrix adaptation evolution strategy that optimizes the Fourier representation of periodic functions entering a range of partial differential equations. We demonstrate the power of the approach by presenting a full catalog of all possible topological bands in two dimensions in the absence of spin-orbit coupling for systems of photons, phonons, electrons, and ultra-cold atoms. Our approach is by no means constrained to the presented application. Generalizations to higher dimensions, systems that break time-reversal symmetry, or further platforms, including those of non-linear systems, should be straightforward to implement. While our focus has been exclusively on platforms with time-reversal and without spin-orbit coupling, our method is rather agnostic towards the specifics of a given system. As long as it can be described by a partial differential equation and takes a set of Fourier coefficients as input, our method is directly applicable. Generalizations to other continuous or discrete parameters are also conceivable with a bit of effort.

## Supplementary Material

pgaf285_Supplementary_Data

## Data Availability

The codes (in python) used for the analysis and visualization are available on the open repository zenodo at the address https://zenodo.org/records/13986212 with the DOI: 10.5281/zenodo.13986212.

## Preprints

This manuscript was posted on a preprint: https://arxiv.org/abs/2410.10484.
