# Supplementary material for: Design and characterization of all 2D fragile topological bands
Source: PNAS Nexus. 2025 Sep 5;4(9):pgaf285. doi: 10.1093/pnasnexus/pgaf285 (PMC12448876; doi:10.1093/pnasnexus/pgaf285)
Supplement: pgaf285_Supplementary_Data [file pgaf285_supplementary_data.pdf]

Supplementary material for: Design and characterization of all two-dimensional fragile topological bands

Samuel Bird,[1,*] Chiara Devescovi,[1,2] Pascal Engeler,[1] Agnes Valenti,[1,3] Doruk Efe Gökmen,[1,4,5] Robin Worreby,[1] Valerio Peri,[6,1] and Sebastian D. Huber[1]

[1] *Institute for Theoretical Physics, ETH Zurich, 8093 Zürich, Switzerland*
[2] *Donostia International Physics Center, Paseo Manuel de Lardizabal 4, 20018 Donostia-San Sebastián, Spain*
[3] *Center for Computational Quantum Physics, Flatiron Institute, New York, NY, 10010, USA*
[4] *James Franck Institute and Department of Statistics, University of Chicago, Chicago, IL 60637, USA*
[5] *National Institute for Theory and Mathematics in Biology, Chicago, IL 60611, USA*
[6] *Department of Physics and Institute of Quantum Information and Matter, California Institute of Technology, Pasadena, CA 91125, USA*

CONTENTS

List of Figures 2

List of Tables 3

I. Considered models 3
A. Mass patterned pre-stressed membrane 3
B. Transverse magnetic modes in a two-dimensional photonic crystal 4
C. Transverse electric modes in a two-dimensional photonic crystal 4
D. Two-dimensional Schrödinger equation 4

II. Algorithm flow chart 5

III. The eleven wallpaper groups with fragile roots 6
A. Generic bundling strategy 6
B. *p*2 7
1. Basic group properties 7
2. Bundling strategy 7
3. Examples 8
C. *p*2*mm* 9
1. Basic group properties 9
2. Bundling strategy 12
3. Examples 12
D. *c*2*mm* 13
1. Basic group properties 13
2. Bundling strategy 16
3. Examples 16
E. *p*4 17
1. Basic group properties 17
2. Bundling strategy 17
3. Examples 19
F. *p*4*mm* 20
1. Basic group properties 20
2. Bundling strategy 20
3. Examples 21
G. *p*4*gm* 25
1. Basic group properties 25

* sabird@phys.ethz.ch

2. Bundling strategy 29
3. Examples 29
H. $p3$ 30
1. Basic group properties 30
2. Bundling strategy 31
3. Examples 31
I. $p3m1$ 32
1. Basic group properties 32
2. Bundling strategy 33
3. Examples 33
J. $p31m$ 34
1. Basic group properties 34
2. Bundling strategy 35
3. Examples 35
K. $p6$ 36
1. Basic group properties 36
2. Bundling strategies 37
3. Examples 38
L. $p6mm$ 42
1. Basic group properties 42
2. Bundling strategies 43
3. Examples 43

References 43

LIST OF FIGURES

S1 Flowchart of structure search algorithm. 5
S2 $p2$ unit cell. 7
S3 $p2$ phonons/TM examples. 9
S4 $p2$ Schrödinger examples. examples. 10
S5 $p2$ TE examples. 11
S6 $p2mm$ unit cell. 12
S7 $p2mm$ phonons/TM examples. 13
S8 $p2mm$ Schrödinger examples. examples. 14
S9 $p2mm$ TE examples. 15
S10 $c2mm$ unit cell. 16
S11 $c2mm$ phonons/TM examples. 17
S12 $c2mm$ Schrödinger examples. 18
S13 $c2mm$ TE examples. 19
S14 $p4$ unit cell. 19
S15 $p4$ phonons/TM examples. 22
S16 $p4$ Schrödinger examples. 23
S17 $p4$ TE examples. 24
S18 $p4gm$ unit cell. 25
S19 $p4mm$ phonons/TM examples. 26
S20 $p4mm$ Schrödinger examples. 27
S21 $p4mm$ TE examples. 28
S22 $p4gm$ unit cell. 29
S23 $p4gm$ bundle linkage. 30
S24 $p4gm$ TE examples. 30
S25 $p3$ unit cell. 31
S26 $p3$ phonons/TM examples. 32
S27 $p3$ Schrödinger examples. 32
S28 $p3$ TE examples. 32
S29 $p3m1$ unit cell. 33
S30 $p3m1$ phonons/TM examples. 34

S31 $p3m1$ Schrödinger examples. 34
S32 $p3m1$ TE examples. 34
S33 $p31m$ unit cell. 35
S34 $p31m$ phonons/TM examples. 36
S35 $p31m$ Schrödinger examples. 36
S36 $p31m$ TE examples. 36
S37 $p6$ unit cell. 37
S38 $p6$ phonons/TM examples. 39
S39 $p6$ Schrödinger examples. 40
S40 $p6$ TE examples. 41
S41 $p6mm$ unit cell. 42
S42 $p6mm$ phonons/TM examples. 44
S43 $p6mm$ Schrödinger examples. 45
S44 $p6mm$ TE examples. 46

LIST OF TABLES

S1 $p2$ irreps. 7
S2 $p2$ roots. 7
S3 $p4$ irreps. 12
S4 $p2mm$ roots. 12
S5 $c2mm$ irreps. 16
S6 $c2mm$ roots. 16
S7 $p4$ irreps. 18
S8 $p4$ roots. 20
S9 $p4mm$ irreps. 20
S10 $p4mm$ roots. 21
S11 $p4gm$ irreps. 25
S12 $p4gm$ roots. 25
S13 $p3$ irreps. 31
S14 $p3$ roots. 31
S15 $p3m1$ irreps. 33
S16 $p3m1$ roots. 33
S17 $p31m$ irreps. 35
S18 $p31m$ root. 35
S19 $p6$ irreps. 37
S20 $p6$ roots. 37
S21 $p6mm$ irreps. 42
S22 $p6mm$ roots. 43

I. CONSIDERED MODELS

In this section, we explain which models we consider in the statistical analysis of the fragile roots in the main text. All of them are described by scalar partial differential equations, where some coefficients are space-dependent with the symmetry given by one of the wallpaper groups. The Poisson equation either describes the transverse displacement of an elastic membrane or the z-component of the electric field for the transverse magnetic (TM) modes of the Maxwell equations in a slab geometry. For the transverse electric (TE) modes, the equation is slightly more complicated, see below. Finally, we consider the Schrödinger equation for a single electron confined to two dimensions exposed to an artificially structured potential.

A. Mass patterned pre-stressed membrane

We consider an elastic membrane with a mass profile $\sigma(x, y)$

$$\nabla^2\phi(x, y) = -\sigma(x, y)\omega^2\phi(x, y). \tag{S1}$$

We parameterize the mass profile $\sigma_{\text{bare}}(x, y)$ with Fourier coefficients $n_1\boldsymbol{G}_1 + n_2\boldsymbol{G}_2$ ($n_1, n_2 \in \mathbb{Z}$), using the reciprocal lattice vectors

$$\boldsymbol{G}_i \cdot \boldsymbol{a}_j = 2\pi\delta_{ij} \tag{S2}$$

of the corresponding lattice spanned by $\boldsymbol{a}_1$ and $\boldsymbol{a}_2$.

To be close to an easily manufacturable structure, e.g., a pre-stressed Silicon nitride membrane with gold patterning to achieve a periodic mass profile, we further use

$$\sigma(x, y) = \frac{\sigma_{\text{max}} - \sigma_{\text{min}}}{1 + e^{-\beta\sigma_{\text{bare}}(x,y)}} + \sigma_{\text{min}}. \tag{S3}$$

Unless otherwise stated we use $|n_i| \leq 4$, $\beta = 2$, $\sigma_{\text{min}} = 5$, and $\sigma_{\text{max}} = 100$. Note, that (S1) is scale invariant under $\sigma \to \lambda\sigma$, and hence the relevant parameter is the dynamical range $\eta = \sigma_{\text{max}}/\sigma_{\text{min}} = 20$.

B. Transverse magnetic modes in a two-dimensional photonic crystal

TM modes two-dimensional photonic crystal are described by [S1]

$$\nabla^2 E_z(x, y) = -\epsilon(x, y)\omega^2 E_z(x, y). \tag{S4}$$

This is formally the same equation as for the membranes above (S1) when identifying $\epsilon \equiv \sigma$.

C. Transverse electric modes in a two-dimensional photonic crystal

TE modes two-dimensional photonic crystal are described by [S1]

$$-\nabla \log \epsilon(x, y) \cdot \nabla H_z(x, y) + \nabla^2 H_z(x, y) = -\epsilon(x, y)\omega^2 H_z(x, y). \tag{S5}$$

We encode the dielectric constant as above.

D. Two-dimensional Schrödinger equation

The two-dimensional Schrödinger equation in a periodic potential is given by

$$\nabla^2\psi(x, y) = [V(x, y) + \omega]\,\psi(x, y). \tag{S6}$$

Note that the Schrödinger equation is *not* scale invariant. The typical kinetic energy of a superstructure defined by $V(x, y)$ is given by

$$E_{\text{kin}} = \frac{\hbar^2}{2m^*}\left(\frac{\pi}{a}\right)^2, \tag{S7}$$

where a is the lattice constant, and m^* the effective band mass.

We highlight two specific applications of Eq. (S6). First electrons confined to two dimensions either in van der Waals materials or, e.g., on the (111) surface of Cu. Here, smaller a are harder to fabricate, larger a lower the temperature scale. For the purpose of this work we use $a \approx 10\,\text{nm}$ and an effective mass of about a third of the electron mass, giving rise to a typical temperature scale of $E_{\text{kin}}/k_{\text{B}} = 1\,\text{K}$. Assuming the ability to expose the electron gas to a potential varying about an electronvolt, we use

$$V(x, y) = \frac{V_0}{1 + e^{-\beta V_{\text{bare}}(x,y)}} \tag{S8}$$

with a $V_0 = 1000$. Note that written like this, we measure the strength of V in units of $E_{\text{natural}} = E_{\text{kin}}/\pi^2$. These numbers are inspired, but not constrained to, carbon monoxide molecules on Cu(111) surfaces [S2]. Another application are cold atoms in optical potentials. There, the established energy scale is

$$E_{\text{recoil}} = \frac{\hbar^2 k^2}{2m} = 16E_{\text{kin}} = 16\pi^2 E_{\text{natural}}, \tag{S9}$$

where m now denotes the mass of the neutral atom and $k = 2\pi/\lambda$, where λ is the wave-length of the light responsible for the potential $V \propto \sin^2(kx)$ with a lattice constant $a = \lambda/2$. In other words, a $V_0 = 1000$ amounts to $\sim 6\, E_{\text{recoil}}$, corresponding to a moderate lattice depth.[1]

II. ALGORITHM FLOW CHART

In Fig.S1, a flow chart outlines the main structure search algorithm used in this paper.

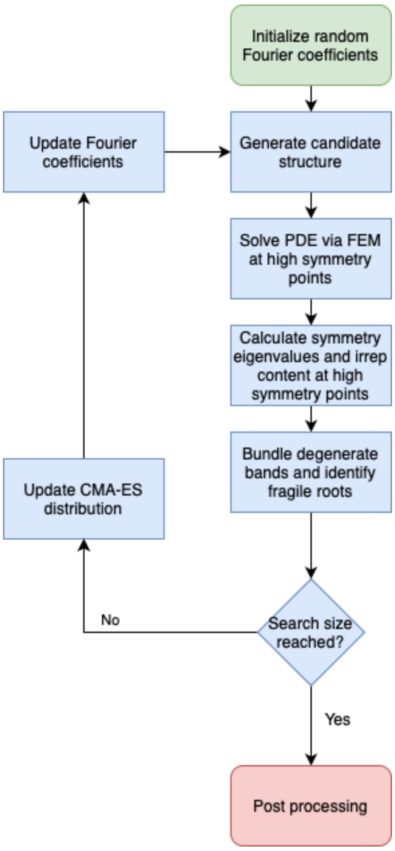

FIG. S1: Flowchart of structure search algorithm.

[1] The superfluid to Mott transition for Rubidium atoms in a square lattice occurs around $13\, E_{\text{recoil}}$ [S3].

III. THE ELEVEN WALLPAPER GROUPS WITH FRAGILE ROOTS

A. Generic bundling strategy

To detect fragile roots, we bundle bands into connected sets (all fragile roots contain at least two bands). When there are no high-symmetry *lines* present in the Brillouin zone, bands cannot generically cross. Hence, it suffices to make use of properties at high-symmetry *points* in the Brillouin zone. Three cases can be separated:

1. Some of the high-symmetry points contain two-dimensional irreducible representations (irreps). By scanning through the irrep content at each high-symmetry point starting from the lowest bands allows us to uniquely determine the band bundles.

2. Some of the high-symmetry points contain conjugate pairs of complex irreps. As we deal with time-reversal symmetric systems, these complex irreps have to come in pairs and hence are equivalent to two-dimensional irreps for the sake of bundling.

3. No conjugate pairs or two-dimensional irreps are present at high-symmetry points. In that case, the irreps alone would only predict singly degenerate bands. However, in all of these cases, such bands would have a Chern number as shown below. Hence, these bands that would have opposite Chern number, if they were isolated, touch somewhere at arbitrary positions in the Brillouin zone. In these cases, one needs to check for all sets of neighboring bands, if by the irreps of the individual bands, one would conclude to have a Chern band, these Chern numbers are rendered zero by bundling them together.

We elaborate on the different scenarios below for each wallpaper group individually. In case there are additional high-symmetry lines induced by mirror or glide-symmetries, bands may be able to cross. When bundling in such groups, one has to take this into account and make sure compatibility relations are fulfilled.

In the following subsections, we provide the symmetries and irreps for all eleven wallpaper groups that contain fragile roots. We discuss the different roots and show one example of a fragile band structure per distinct root.

B. $p2$

1. *Basic group properties*

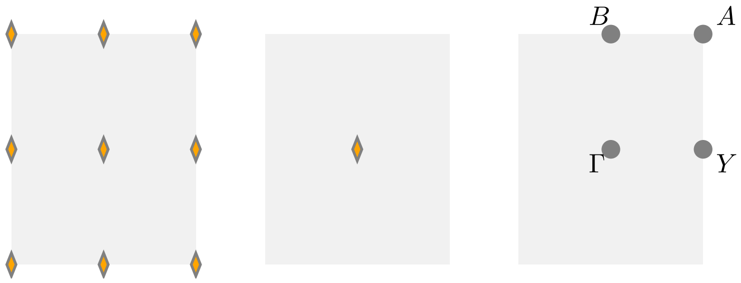

FIG. S2: Left: Unit cell with the full symmetry of $p2$. Middle: General positions of $p2$. Right: Brillouin zone with the high-symmetry points and lines indicated.

The group $p2$ describes a rectangular lattice with lattice vectors $\boldsymbol{a}_1 = (1,0)$ and $\boldsymbol{a}_2 = (0,\beta)$. The corresponding space group is $P2$, (#3) constrained to the x-z plane. The group $p2$ contains the following group elements [retrieve from Bilbao server]

$$\{1|n_1\boldsymbol{a}_1 + n_2\boldsymbol{a}_2\}, \quad \text{with} \quad n_1, n_2 \in \mathbb{Z} \tag{S10}$$
$$\{2|(0,0)\}. \tag{S11}$$

The relevant members of the little groups of the high-symmetry points and lines and their irreps are given in Tab. S1. The full set can be obtained from the Bilbao Server.

Γ

	$\{2\|(0,0)\}$
Γ_1	1
Γ_2	-1

B

	$\{2\|(0,0)\}$
B_1	1
B_2	-1

Y

	$\{2\|(0,0)\}$
Y_1	1
Y_2	-1

A

	$\{2\|(0,0)\}$
A_1	1
A_2	-1

TABLE S1: Irreducible representations of $p2$ at the high-symmetry points $\Gamma = (0,0)$, $B = (0,1/2)$, and $Y = (1/2,0)$ and $A = (1/2,1/2)$.

The fragile roots in $p2$ [S4] are given in Tab. S2; the elementary band representations can be retrieved from the Bilbao server.

#	root	# of bands	type
1	$2A_2 + 2B_1 + 2\Gamma_2 + 2Y_2$	2	Chern
2	$2A_2 + 2B_1 + 2\Gamma_1 + 2Y_1$	2	Chern
3	$2A_1 + 2B_2 + 2\Gamma_2 + 2Y_2$	2	Chern
4	$2A_2 + 2B_2 + 2\Gamma_2 + 2Y_1$	2	Chern
5	$2A_2 + 2B_2 + 2\Gamma_1 + 2Y_2$	2	Chern
6	$2A_1 + 2B_1 + 2\Gamma_2 + 2Y_1$	2	Chern
7	$2A_1 + 2B_2 + 2\Gamma_1 + 2Y_1$	2	Chern
8	$2A_1 + 2B_1 + 2\Gamma_1 + 2Y_2$	2	Chern

TABLE S2: Fragile roots and their types in wallpaper group $p2$

2. *Bundling strategy*

We analyze the structure of the roots in the wallpaper group $p2$ as shown in Tab. S2. As we will see, we need to invoke the vanishing Chern number argument to argue for the bundling of bands into fragile roots. For this, we state

equation for the Chern number C in C_2–symmetric systems [S5, S6]

$$(-1)^C = \prod_{i\,\in\,\mathrm{occ.}} \zeta_i(\Gamma)\zeta_i(B)\zeta_i(Y)\zeta_i(A), \tag{S12}$$

where ζ are eigenvalues of $\{2|(0,0)\}$. Note, that there are no two-dimensional irreps at the high-symmetry points, nor conjugate pairs of complex irreps. Let us consider root #1 as an example. It is easy to see, that if we were to have two disconnected sets of bands

$$(A_2 + B_1 + \Gamma_2 + Y_2) \oplus (A_2 + B_1 + \Gamma_2 + Y_2), \tag{S13}$$

The Fu-Kane formula (S12) would tell us that we have two bands, each with an odd Chern number. Given that we do not break time-reversal symmetry, this cannot happen, and hence, these two sets of bands not touch somewhere off the high-symmetry points. The eight different roots in $p2$ are obtained by a permutation of the irreps. Finally, the types of the roots of $p2$ are indicated in the last column of Tab. S2.

3. Examples

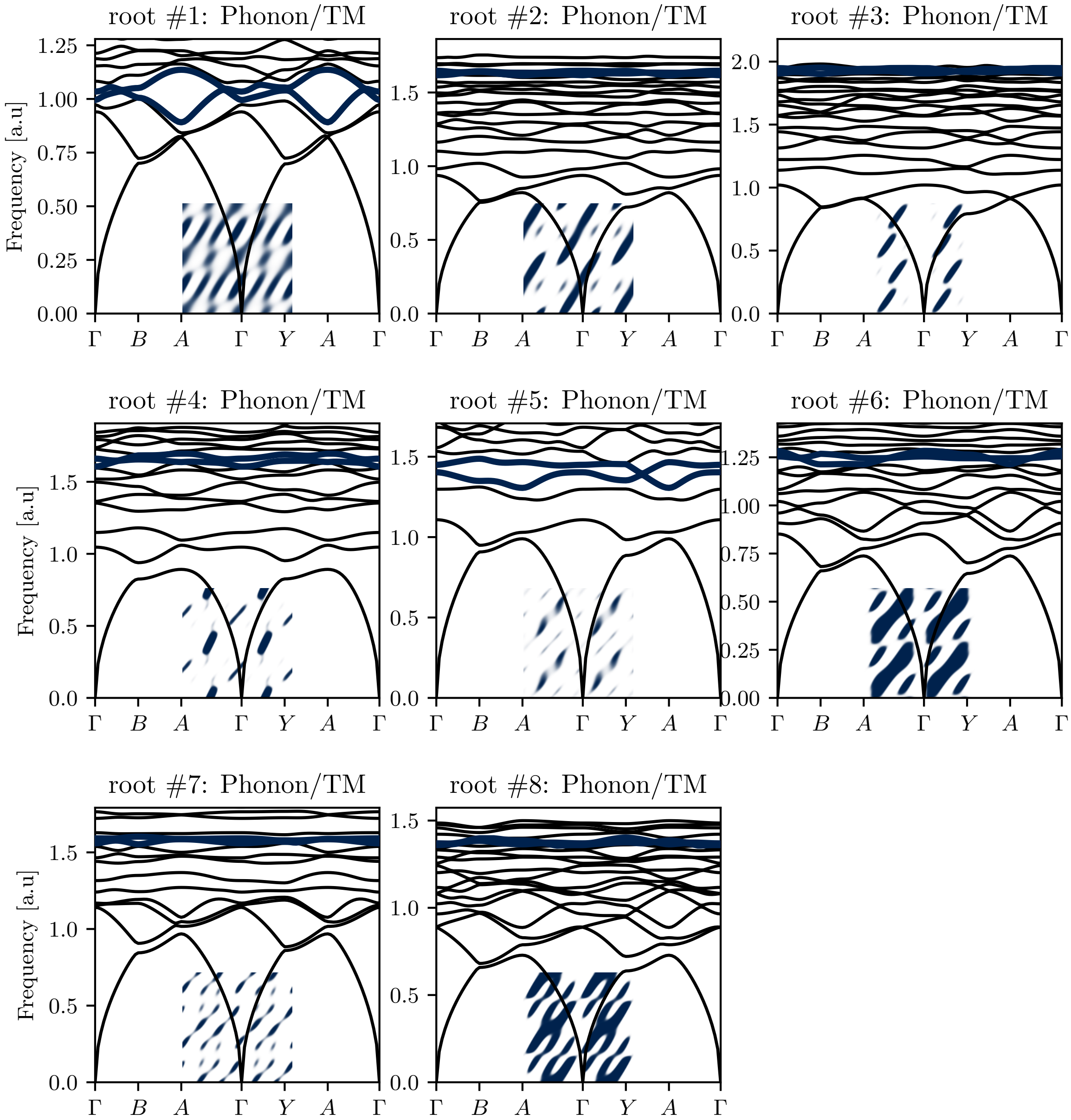

FIG. S3: One sample for each of the roots in $p2$ for phonons and TM photons.

C. *p2mm*

1. *Basic group properties*

The group $p2mm$ describes a rectangular lattice with lattice vectors $\boldsymbol{a}_1 = (1, 0)$ and $\boldsymbol{a}_2 = (0, \beta)$. The corresponding space group is $P2mm$, (#25) constrained to the x-y plane. The group $p2mm$ contains the following group elements

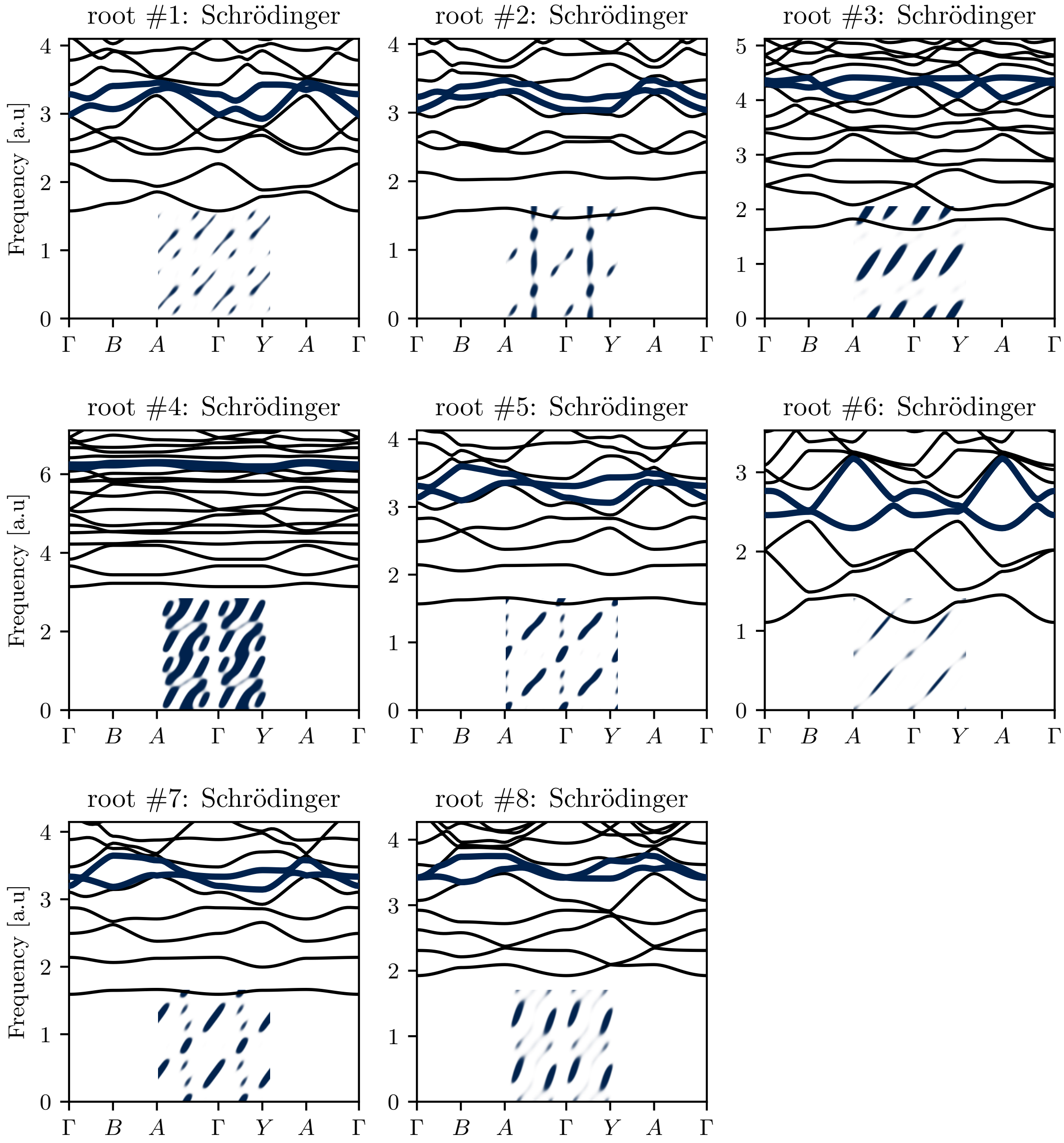

FIG. S4: One sample for each of the roots in $p2$ for systems described by the Schrödinger equation.

[retrieve from Bilbao server]

$$\{1|n_1\boldsymbol{a}_1 + n_2\boldsymbol{a}_2\}, \quad \text{with} \quad n_1, n_2 \in \mathbb{Z} \tag{S14}$$
$$\{2|(0,0)\}, \tag{S15}$$
$$\{m_{10}|(0,0)\},\ \{m_{01}|(0,0)\}. \tag{S16}$$

The relevant members of the little groups of the high-symmetry points and lines and their irreps are given in Tab. S3.

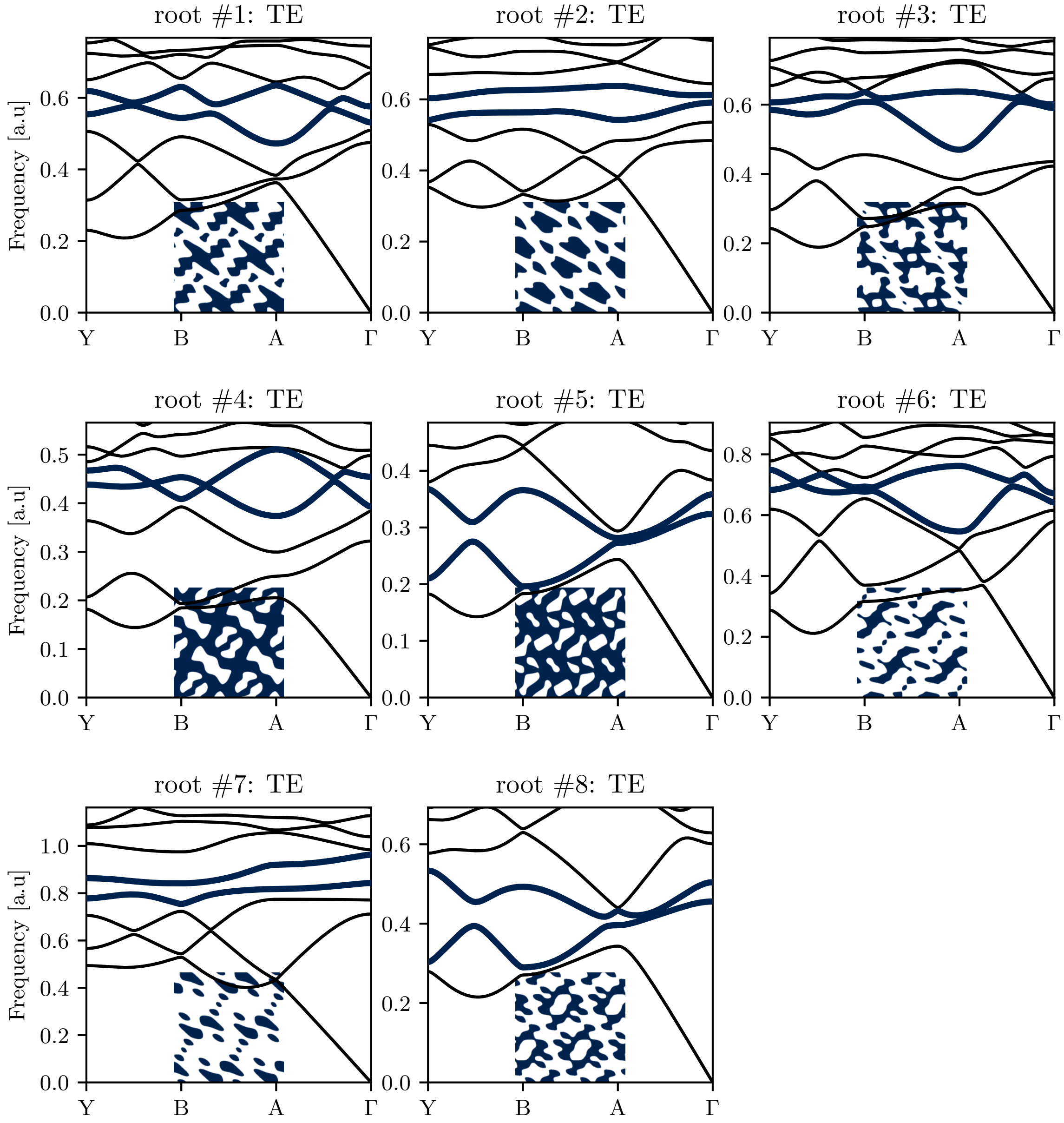

FIG. S5: One sample for each of the roots in $p2$ for TE photons.

The full set can be obtained from the Bilbao Server.
The fragile roots in $p4mm$ [S4] are given in Tab. S4; the elementary band representations can be retrieved from the Bilbao server.

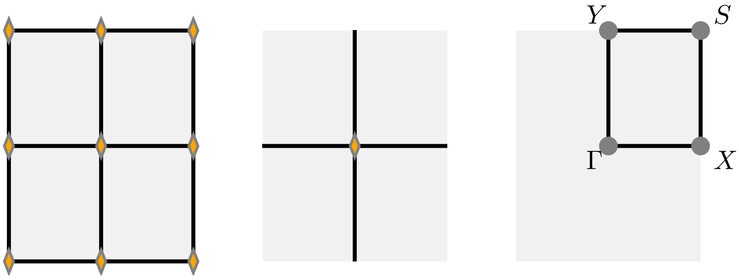

FIG. S6: Left: Unit cell with the full symmetry of $p2mm$. Middle: General positions of $p2mm$. Right: Brillouin zone with the high-symmetry points and lines indicated.

$\Omega = \Gamma,\, X,\, Y,\, S$

	$\{2\|(0,0)\}$	$\{m_{01}\|(0,0)\}$	$\{m_{10}\|(0,0)\}$
Ω_1	1	1	1
Ω_2	1	-1	-1
Ω_3	-1	-1	1
Ω_4	-1	1	-1

$\overline{\Gamma X}$, $\overline{YS}$

	$\{m_{01}\|(0,0)\}$
SM_1	1
SM_2	-1

$\overline{\Gamma Y}$, $\overline{YS}$

	$\{m_{10}\|(0,0)\}$
DT_1	1
DT_2	-1

TABLE S3: The relevant irreducible representations of $p2mm$ at the high-symmetry points $\Gamma = (0,0)$, $X = (1/2,0)$, and $Y = (0,1/2)$ and $S = (1/2,1/2)$ as well as along the lines $\overline{\Gamma X}$, $\overline{\Gamma Y}$, $\overline{XS}$, and $\overline{YS}$.

2. *Bundling strategy*

When consulting Tab. S4, we immediately observe that at each high-symmetry point, the irreps appear exclusively in the combination $\Omega_1 + \Omega_2$ or $\Omega_3 + \Omega_4$ (with $\Omega = \Gamma,\, X,\, Y,\, S$), i.e., with irreps of the same parity, see Tab. S3. Using the same argument as for the group $p2$, we conclude that we again have a Chern number if we were to divide this root into two independent sets of bands. However, the mirrors give us a simpler handle on the bundling.

Let us consider root #1. We start from Γ_1 and go along $\overline{\Gamma X}$, compatibility with the eigenvalues of $\{m_{01}|(0,0)\}$ require us to connect Γ_1–SM_1–X_4. Continuing along $\overline{XS}$ we find X_4–DT_2–S_2 due to the odd eigenvalues of $\{m_{10}|(0,0)\}$. Going along $\overline{YS}$ fixes S_2–SM_2–Y_2, and finally back to Γ via Y_2–DT_2–Γ_2. In other words, we need to traverse the loop Γ–X–S–Y–Γ twice to come back to the irrep Γ_1: The two Chern bands are linked through gap closings along the high-symmetry lines. This means, we can bundle by using the mirror eigenvalues along these lines and can then check for the irrep-content in the so-obtained bundle. There is no need to check the Chern number parity of adjacent bands in $p2mm$. All other roots are permutations of the irreps of root #1. Finally, the types of the roots of $p2mm$ are indicated in the last column of Tab. S4.

3. *Examples*

#	root	# of bands	type
1	$\Gamma_1 + \Gamma_2 + S_1 + S_2 + X_3 + X_4 + Y_1 + Y_2$	2	mirrors
2	$\Gamma_1 + \Gamma_2 + S_3 + S_4 + X_1 + X_2 + Y_1 + Y_2$	2	mirrors
3	$\Gamma_3 + \Gamma_4 + S_3 + S_4 + X_3 + X_4 + Y_1 + Y_2$	2	mirrors
4	$\Gamma_1 + \Gamma_2 + S_3 + S_4 + X_3 + X_4 + Y_3 + Y_4$	2	mirrors
5	$\Gamma_1 + \Gamma_2 + S_1 + S_2 + X_1 + X_2 + Y_3 + Y_4$	2	mirrors
6	$\Gamma_3 + \Gamma_4 + S_3 + S_4 + X_1 + X_2 + Y_3 + Y_4$	2	mirrors
7	$\Gamma_3 + \Gamma_4 + S_1 + S_2 + X_3 + X_4 + Y_3 + Y_4$	2	mirrors
8	$\Gamma_3 + \Gamma_4 + S_1 + S_2 + X_1 + X_2 + Y_1 + Y_2$	2	mirrors

TABLE S4: Fragile roots and their types in wallpaper group $p2mm$

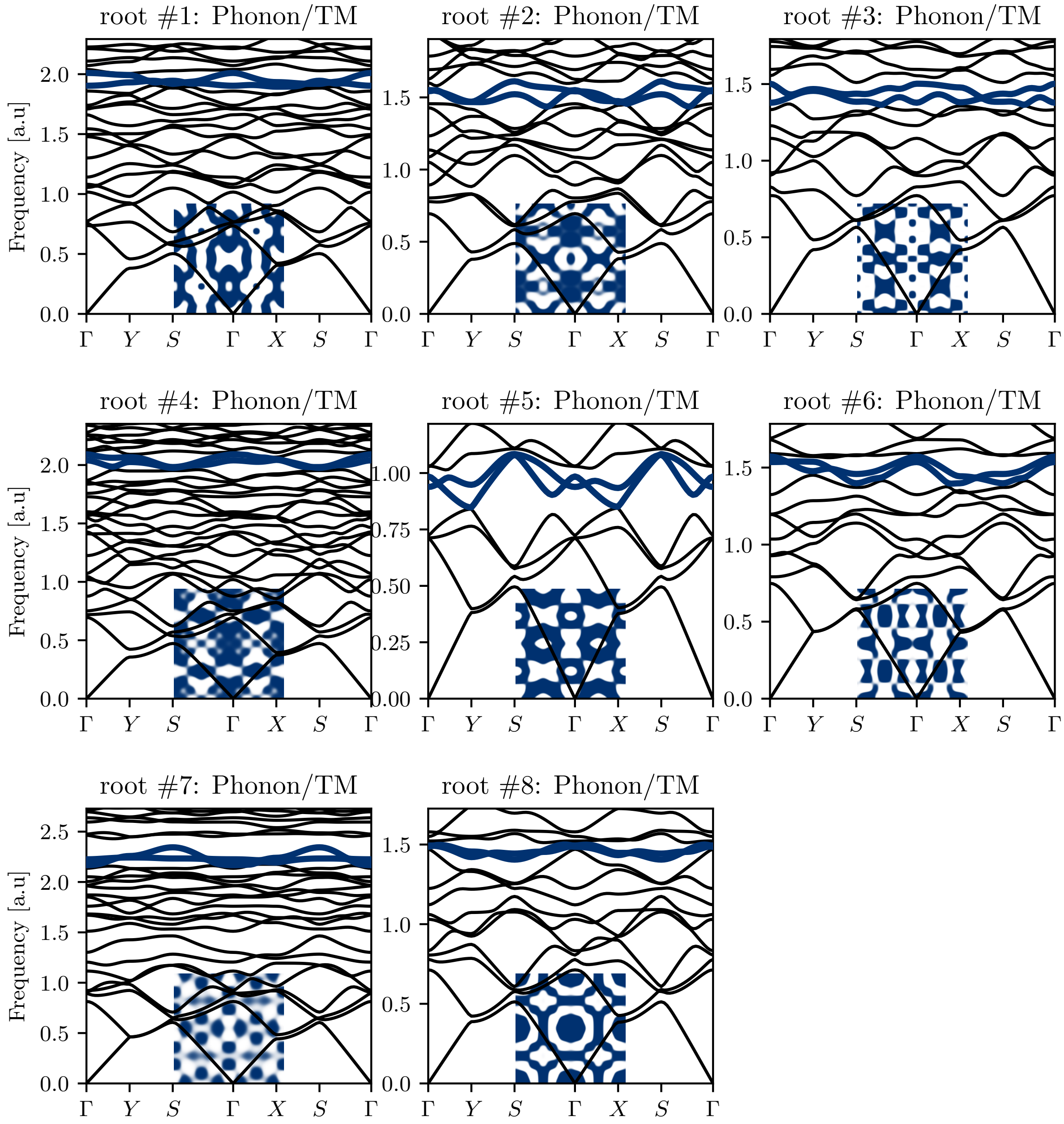

FIG. S7: One sample for each of the roots in $p2mm$ for phonons and TM photons.

D. $c2mm$

1. *Basic group properties*

The group $c2mm$ describes a rhombic lattice with lattice vectors $\boldsymbol{a}_1 = (1, 0)$ and $\boldsymbol{a}_2 = (0, \beta)$. The corresponding space group is $Cmm2$, (#35) constrained to the x-y plane. The group $c2mm$ contains the following group elements

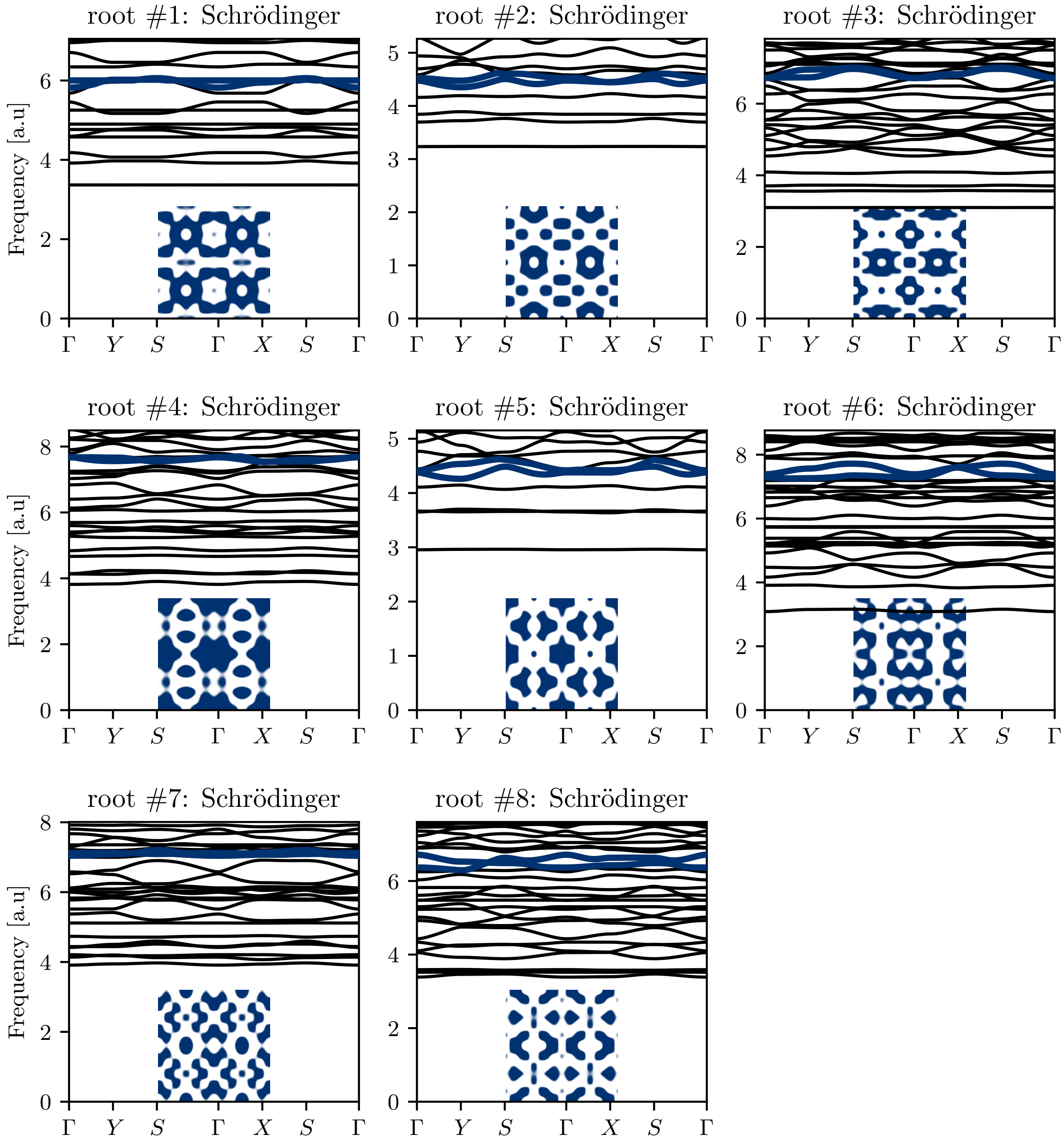

FIG. S8: One sample for each of the roots in $p2mm$ for systems described by the Schrödinger equation.

[retrieve from Bilbao server]

$$\{1|n_1\boldsymbol{a}_1 + n_2\boldsymbol{a}_2\}, \quad \text{with} \quad n_1, n_2 \in \mathbb{Z} \tag{S17}$$

$$\{2|(0,0)\}, \tag{S18}$$

$$\{m_{01}|(0,0\}, \ \{m_{10}|(0,0)\}. \tag{S19}$$

The relevant members of the little groups of the high-symmetry points and lines and their irreps are given in Tab. S5.

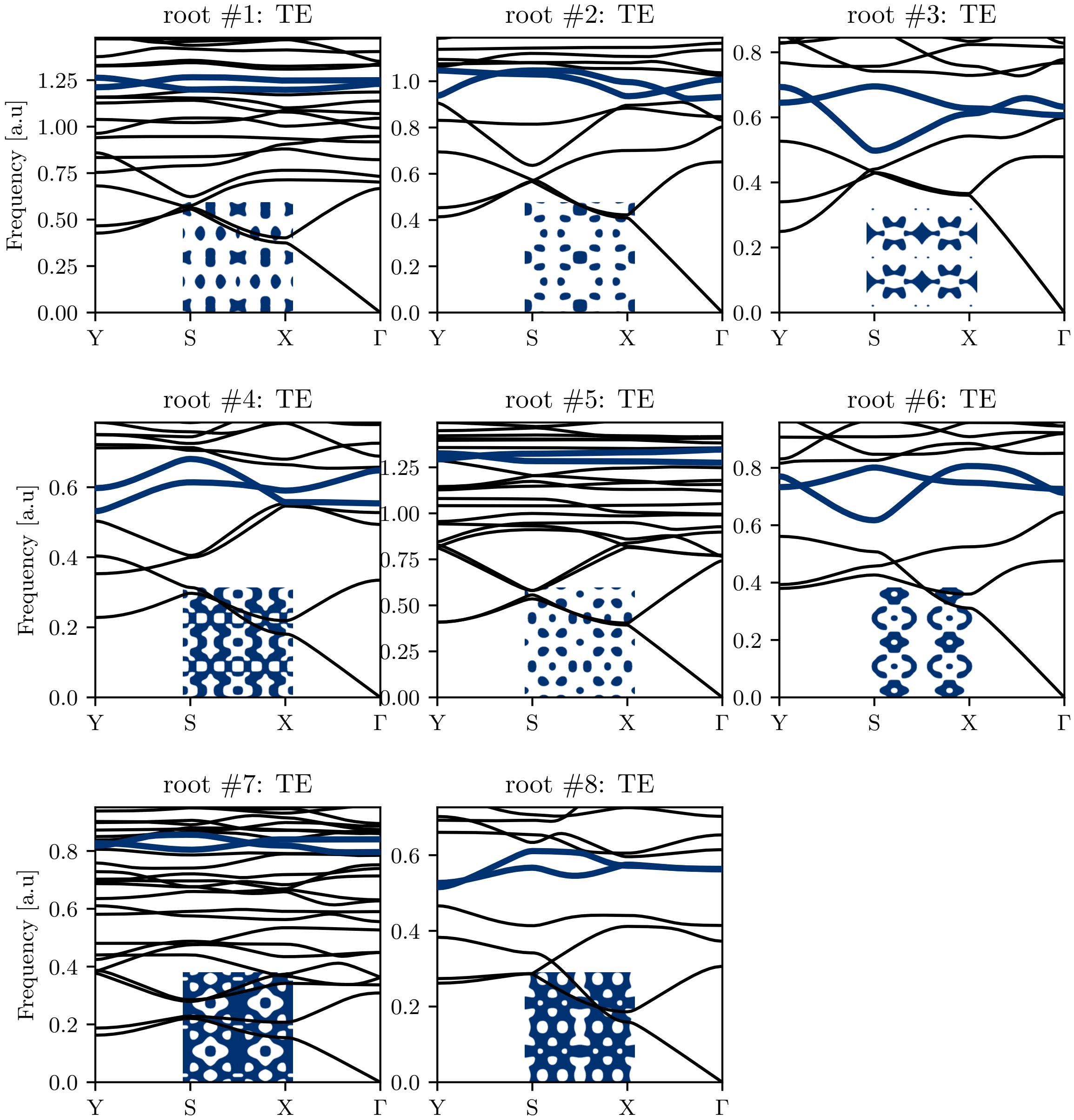

FIG. S9: One sample for each of the roots in $p2mm$ for TE photons.

The full set can be obtained from the Bilbao Server.
The fragile roots in $c2mm$ [S4] are given in Tab. S6; the elementary band representations can be retrieved from the Bilbao server.

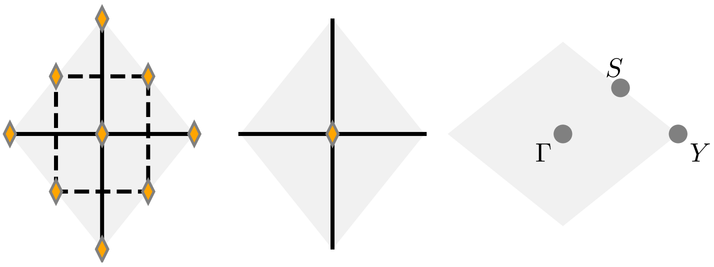

FIG. S10: Left: Unit cell with the full symmetry of $c2mm$. Middle: General positions of $c2mm$. Right: Brillouin zone with the high-symmetry points and lines indicated.

$\Omega = \Gamma, Y$

	$\{2\|(0,0)\}$	$\{m_{01}\|(0,0)\}$	$\{m_{10}\|(0,0)\}$
Ω_1	1	1	1
Ω_2	1	-1	-1
Ω_3	-1	-1	1
Ω_4	-1	1	-1

S

	$\{2\|(0,0)\}$
S_1	1
S_2	-1

TABLE S5: The relevant irreducible representations of $c2mm$ at the high-symmetry points $\Gamma = (0,0)$, $Y = (1,0)$, and $S = (1/2, 1/2)$.

2. *Bundling strategy*

As is obvious from the irreps at Γ, Y, and S, there are no 2D irreps, nor conjugate pairs. Moreover, no mirror symmetric lines are present in the Brillouin zone due to the rhombic lattice structure. We are therefore left with a Chern argument.

Let us inspect root #1: Given that Γ_1 and Γ_2 have the parity as much as Y_3 and Y_4, it does not matter which of the two we connect. The Fu-Kane formula (S12) immediately yields an odd Chern number: We need the two bands so constructed to cross somewhere in the Brillouin zone the yield a time-reversal symmetric bundle. Consequently, we apply the Chern bundling strategy outlined above. Finally, the types of the roots of $p2$ are indicated in the last column of Tab. S6.

3. *Examples*

#	root	# of bands	type
1	$\Gamma_1 + \Gamma_2 + Y_3 + Y_4 + 2S_2$	2	Chern
2	$\Gamma_1 + \Gamma_2 + Y_3 + Y_4 + 2S_1$	2	Chern
3	$\Gamma_3 + \Gamma_4 + Y_1 + Y_2 + 2S_2$	2	Chern
4	$\Gamma_3 + \Gamma_4 + Y_1 + Y_2 + 2S_1$	2	Chern

TABLE S6: Fragile roots and their types in wallpaper group $c2mm$

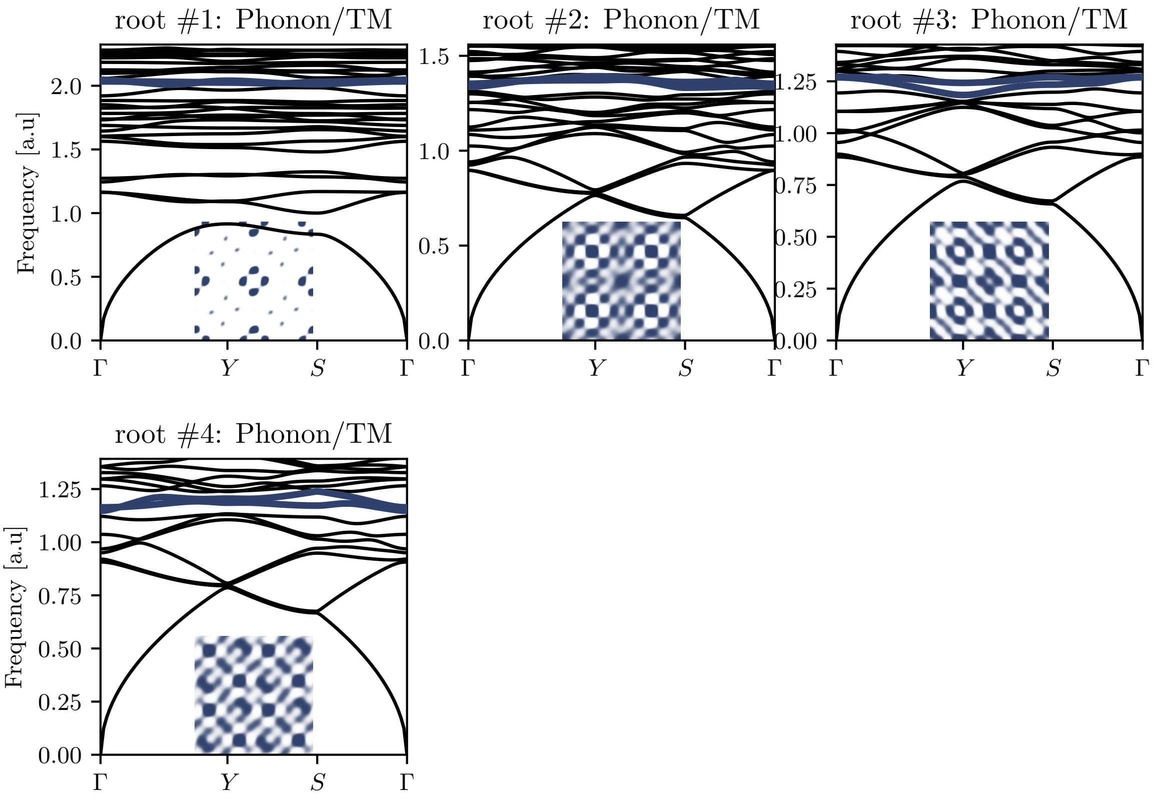

FIG. S11: One sample for each of the roots in $c2mm$ for phonons and TM photons.

E. $p4$

1. Basic group properties

The group $p4$ describes a square lattice with lattice vectors $\boldsymbol{a}_1 = (1,0)$ and $\boldsymbol{a}_2 = (0,1)$. The corresponding space group is $P4$, (#75) constrained to the x-y plane. The group $p4$ contains the following group elements [retrieve from Bilbao server]

$$\{1|n_1\boldsymbol{a}_1 + n_2\boldsymbol{a}_2\}, \quad \text{with} \quad n_1, n_2 \in \mathbb{Z} \tag{S20}$$

$$\{2|(0,0)\},\ \{4^+|(0,0)\},\ \{4^-|(0,0)\},. \tag{S21}$$

$$\tag{S22}$$

The relevant members of the little groups of the high-symmetry points and lines and their irreps are given in Tab. S7. The full set can be obtained from the Bilbao Server.
The fragile roots in $p4$ [S4] are given in Tab. S8; the elementary band representations can be retrieved from the Bilbao server.

2. Bundling strategy

We start by addressing roots #1-#3 and #5 as these do not involve any conjugate pairs of irreps ($p4$ does not have any two-dimensional irreps). As in $p2$, we will rely on a Chern number argument. We state the following formula for

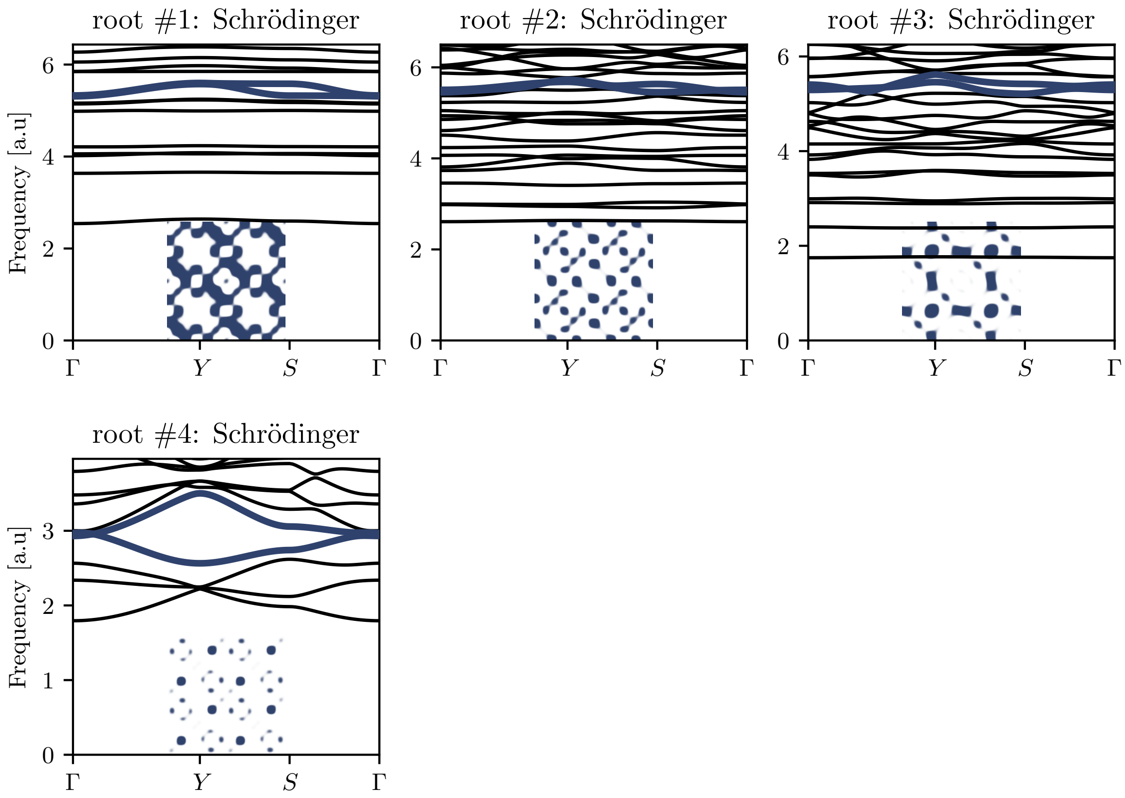

FIG. S12: One sample for each of the roots in $c2mm$ for systems described by the Schrödinger equation.

Γ

	$\{2\|(0,0)\}$	$\{4^+\|(0,0)\}$
Γ_1	1	1
Γ_2	1	−1
Γ_3	−1	i
Γ_4	−1	−i

M

	$\{2\|(0,0)\}$	$\{4^+\|(0,0)\}$
M_1	1	1
M_2	1	−1
M_3	−1	i
M_4	−1	−i

X

	$\{2\|(0,0)\}$
X_1	1
X_2	−1

TABLE S7: The relevant irreducible representations of $p4$ at the high-symmetry points $\Gamma = (0,0)$, $M = (1/2, 1/2)$, and $X = (0, 1/2)$.

the Chern number C in C_4–symmetric systems [S6]

$$\mathrm{i}^C = \prod_{i\,\in\,\mathrm{occ.}} (-1)^F \xi_i(\Gamma)\xi_i(M)\zeta_i(X), \tag{S23}$$

where $F = 2S$, with S the total spin of the particles, ξ and ζ the eigenvalues of $\{4^+|(0,0)\}$ and $\{2|(0,0)\}$, respectively. In the following we set $F = 0$.

We see that root #1 can be written as

$$(\Gamma_2 + M_2 + X_2) \oplus (\Gamma_2 + M_2 + X_2). \tag{S24}$$

Using (S23), the above two isolated bands would each have a Chern number

$$C = 2 + 4n \quad \text{with} \quad n \in \mathbb{Z}. \tag{S25}$$

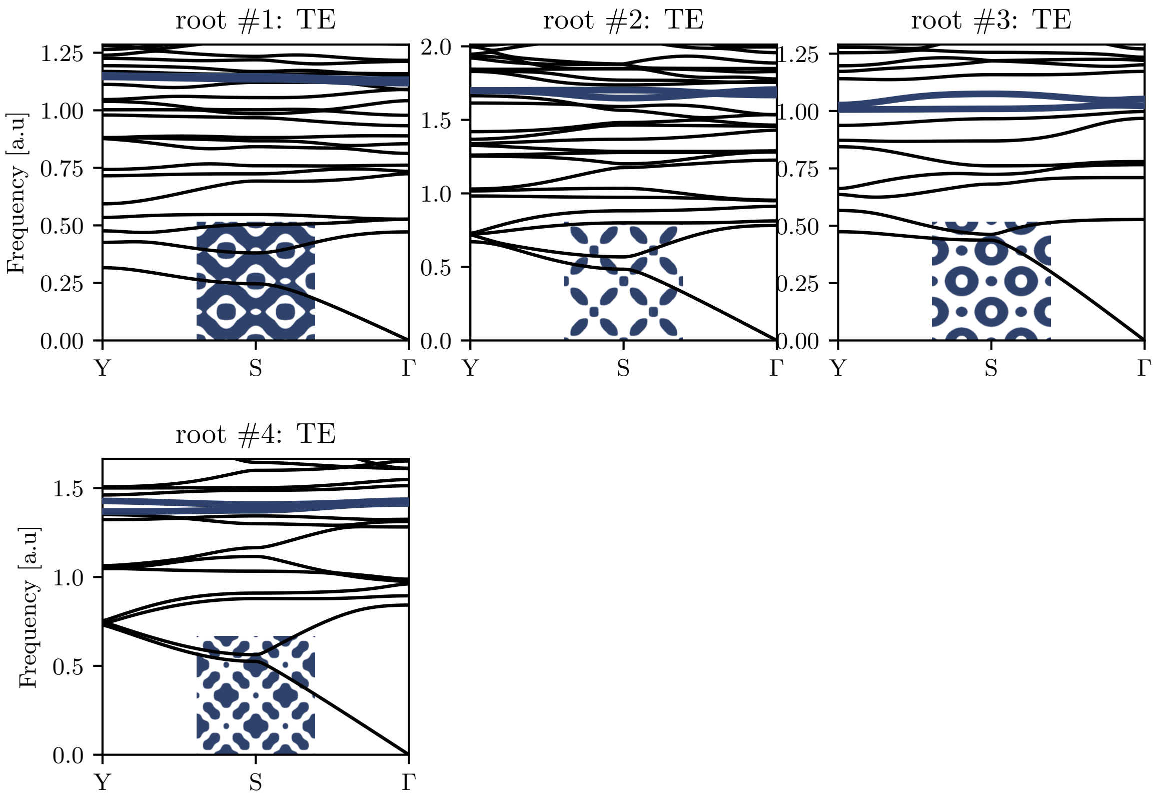

FIG. S13: One sample for each of the roots in $c2mm$ for TE photons.

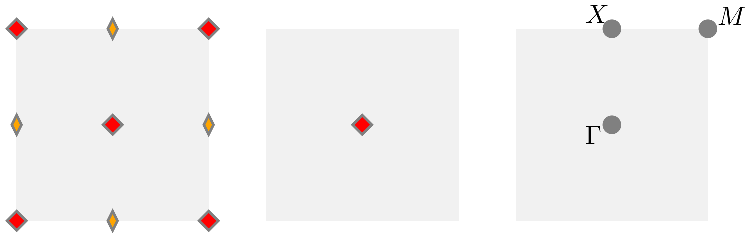

FIG. S14: Left: Unit cell with the full symmetry of $p4$. Middle: General positions of $p4$. Right: Brillouin zone with the high-symmetry points and lines indicated.

To be compliant with time-reversal symmetry, these two bands therefore need to be connected somewhere in the Brillouin zone. The remaining roots all contain a pair of conjugate irreps, which glues them into a bundle of two. Finally, the types of the roots of $p4$ are indicated in the last column of Tab. S8.

3. Examples

#	root	# of bands	type
1	$2\Gamma_2+2M_2+2X_2$	2	Chern
2	$2\Gamma_1+2M_2+2X_1$	2	Chern
3	$2\Gamma_2+2M_1+2X_1$	2	Chern
4	$2\Gamma_1+M_3M_4+2X_2$	2	conjugate pairs
5	$2\Gamma_1+2M_1+2X_2$	2	Chern
6	$2\Gamma_1+M_3M_4+2X_1$	2	conjugate pairs
7	$2\Gamma_2+M_3M_4+2X_1$	2	conjugate pairs
8	$2\Gamma_2+M_3M_4+2X_2$	2	conjugate pairs
9	$\Gamma_3\Gamma_4+2M_2+2X_2$	2	conjugate pairs
10	$\Gamma_3\Gamma_4+2M_1+2X_1$	2	conjugate pairs
11	$\Gamma_3\Gamma_4+2M_2+2X_1$	2	conjugate pairs
12	$\Gamma_3\Gamma_4+2M_1+2X_2$	2	conjugate pairs

TABLE S8: Fragile roots and their types in wallpaper group $p4$.

F. *p4mm*

1. *Basic group properties*

The group $p4mm$ describes a square lattice with lattice vectors $\boldsymbol{a}_1=(1,0)$ and $\boldsymbol{a}_2=(0,1)$. The corresponding space group is $P4mm$, (#99) constrained to the x-y plane. The group $p4gm$ contains the following group elements [retrieve from Bilbao server]

$$\{1|n_1\boldsymbol{a}_1+n_2\boldsymbol{a}_2\},\quad \text{with}\quad n_1,n_2\in\mathbb{Z} \tag{S26}$$

$$\{2|(0,0)\},\ \{4^+|(0,0)\},\ \{4^-|(0,0)\}, \tag{S27}$$

$$\{m_{01}|(0,0)\},\ \{m_{10}|(0,0)\},\ \{m_{11}|(0,0)\},\ \{m_{1\bar{1}}|(0,0)\}. \tag{S28}$$

The relevant members of the little groups of the high-symmetry points and lines and their irreps are given in Tab. S9. The full set can be obtained from the Bilbao Server.

$\Omega=\Gamma,\ M$

	$\{2\|(0,0)\}$	$\{4^+\|(0,0)\}$	$\{m_{10}\|(0,0)\}$	$\{m_{01}\|(0,0)\}$	$\{m_{1\bar{1}}\|(0,0)\}$
Ω_1	1	1	1	1	1
Ω_2	1	-1	1	1	-1
Ω_3	1	-1	-1	-1	1
Ω_4	1	1	-1	-1	-1
Ω_5	$-\mathbb{1}$	$-\mathrm{i}\sigma_y$	$-\sigma_x$	σ_x	$-\sigma_z$

X

	$\{2\|(0,0)\}$	$\{m_{10}\|(0,0)\}$	$\{m_{01}\|(0,0)\}$
X_1	1	1	1
X_2	1	-1	-1
X_3	-1	1	-1
X_4	-1	-1	1

$\overline{\Gamma X}$

	$\{m_{10}\|(0,0)\}$
DT_1	1
DT_2	-1

$\overline{\Gamma M}$

	$\{m_{1\bar{1}}\|(0,0)\}$
SM_1	1
SM_2	-1

$\overline{XM}$

	$\{m_{01}\|(0,0)\}$
Y_1	1
Y_2	-1

TABLE S9: The relevant irreducible representations of $p4mm$ at the high-symmetry points $\Gamma=(0,0)$, $M=(1/2,1/2)$, and $X=(0,1/2)$ as well as along the lines $\overline{\Gamma X}$, $\overline{\Gamma M}$, and $\overline{XM}$.

The fragile roots in $p4mm$ [S4] are given in Tab. S10; the elementary band representations can be retrieved from the Bilbao server.

2. *Bundling strategy*

We start with the roots #1, #4, #5, #8–#12. All of those contain either Γ_5 or M_5, i.e., a two-dimensional irrep. The mirror eigenvalues along the lines connecting these points to their partners fully determine the bundling.

#	root	# of bands	type
1	$\Gamma_2+\Gamma_3+M_5+X_3+X_4$ 2D	2	2D irrep & mirrors
2	$\Gamma_2+\Gamma_3+M_2+M_3+X_3+X_4$	2	mirrors
3	$\Gamma_1+\Gamma_4+M_1+M_4+X_3+X_4$	2	mirrors
4	$\Gamma_1+\Gamma_4+M_5+X_1+X_2$	2	2D irrep & mirrors
5	$\Gamma_2+\Gamma_3+M_5+X_1+X_2$	2	2D irrep & mirrors
6	$\Gamma_2+\Gamma_3+M_1+M_4+X_1+X_2$	2	mirrors
7	$\Gamma_1+\Gamma_4+M_2+M_3+X_1+X_2$	2	mirrors
8	$\Gamma_5+M_1+M_4+X_3+X_4$	2	2D irrep & mirrors
9	$\Gamma_5+M_2+M_3+X_1+X_2$	2	2D irrep & mirrors
10	$\Gamma_5+M_2+M_3+X_3+X_4$	2	2D irrep & mirrors
11	$\Gamma_5+M_1+M_4+X_1+X_2$	2	2D irrep & mirrors
12	$\Gamma_1+\Gamma_4+M_5+X_3+X_4$	2	2D irrep & mirrors

TABLE S10: Fragile roots and their types in wallpaper group $p4mm$.

For the remaining root #2, #3, #6, #7 the situation is the same as of $p2mm$: The Chern number formula (S23) suggests that if we were to divide these two sets of bands into disconnected sets, we were to have a Chern number. However, here again, mirrors come to our rescue: Starting from Γ_2 of root #2, we need to traverse the loop Γ–M–X–Γ twice to come back to Γ_2. In other words, the mirror eigenvalues force a bundle of two bands. Finally, the types of the roots of $p4mm$ are indicated in the last column of Tab. S10.

3. Examples

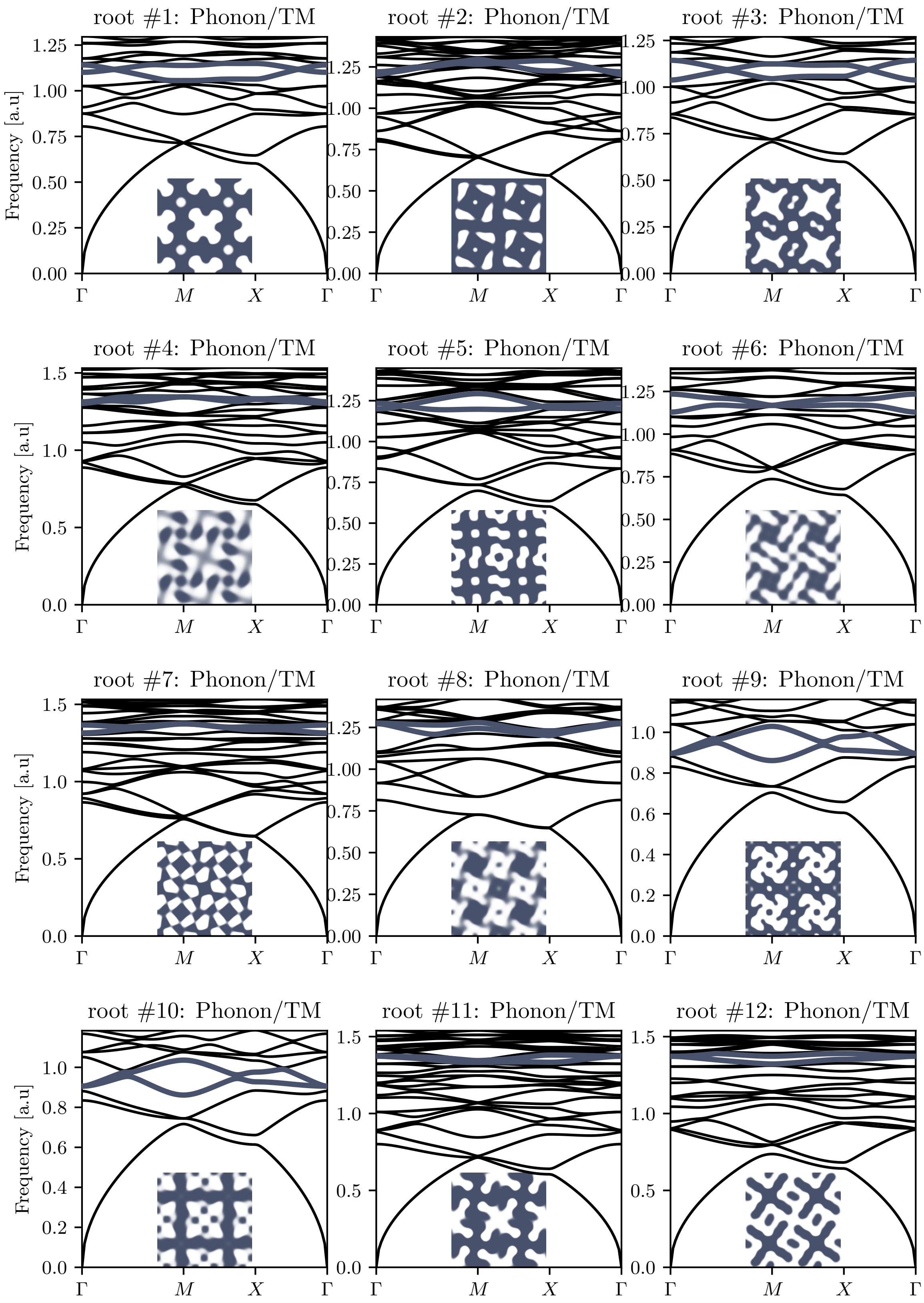

FIG. S15: One sample for each of the roots in $p4$ for phonons and TM photons.

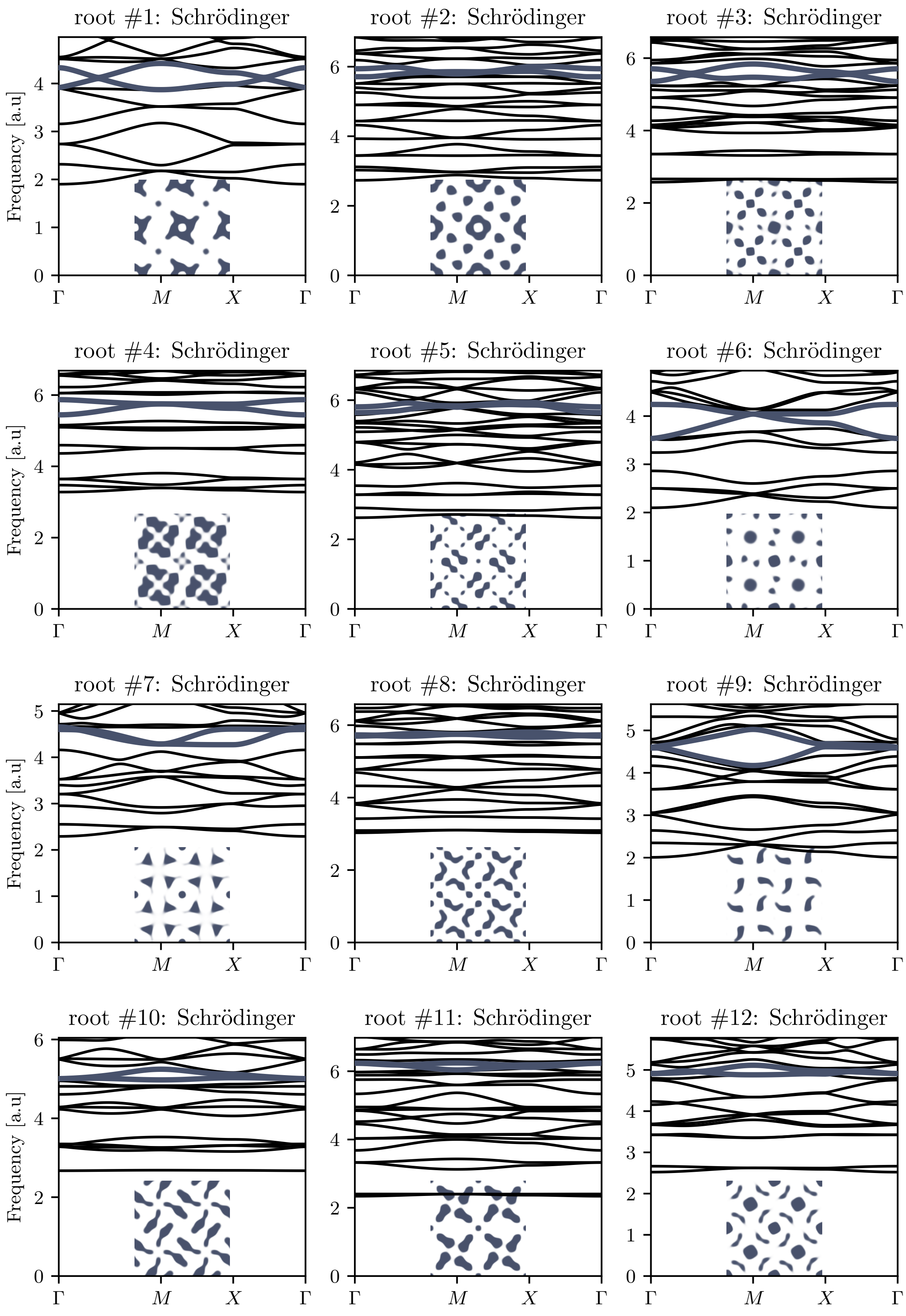

FIG. S16: One sample for each of the roots in $p4$ for systems described by the Schrödinger equation.

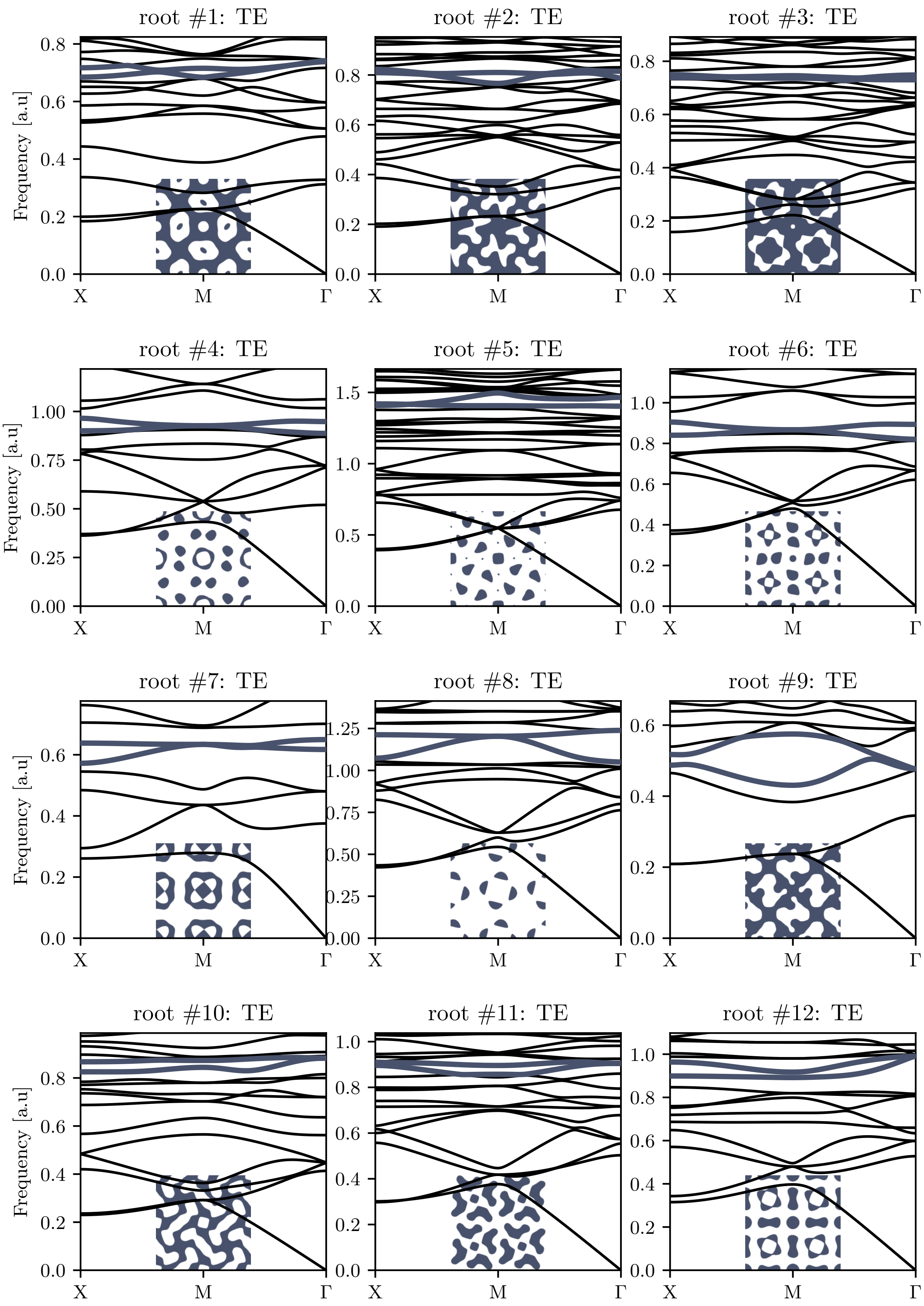

FIG. S17: One sample for each of the roots in $p4$ for TE photons.

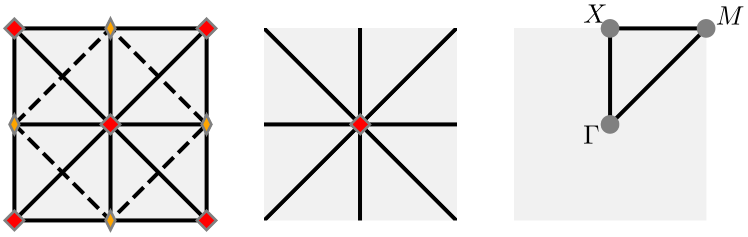

FIG. S18: Left: Unit cell with the full symmetry of $p4mm$. Middle: General positions of $p4mm$. Right: Brillouin zone with the high-symmetry points and lines indicated.

G. $p4gm$

1. Basic group properties

The group $p4gm$ describes a square lattice with lattice vectors $\boldsymbol{a}_1 = (1, 0)$ and $\boldsymbol{a}_2 = (0, 1)$. The corresponding space group is $P4bm$, (#100) constrained to the x-y plane. The group $p4gm$ contains the following group elements [retrieve from Bilbao server]

$$\{1|n_1\boldsymbol{a}_1 + n_2\boldsymbol{a}_2\}, \quad \text{with} \quad n_1, n_2 \in \mathbb{Z} \tag{S29}$$

$$\{2|(0,0)\},\ \{4^+|(0,0)\},\ \{4^-|(0,0)\}, \tag{S30}$$

$$\{m_{01}|(1/2,1/2)\},\ \{m_{10}|(1/2,1/2)\},\ \{m_{11}|(1/2,1/2)\},\ \{m_{1\bar{1}}|(1/2,1/2)\}. \tag{S31}$$

The relevant members of the little groups of the high-symmetry points and lines and their irreps are given in Tab. S11. The full set can be obtained from the Bilbao Server.

Γ

	$\{2\|(0,0)\}$	$\{4^+\|(0,0)\}$	$\{m_{01}\|(1/2,1/2)\}$	$\{m_{1\bar{1}}\|(1/2,1/2)\}$
Γ_1	1	1	1	1
Γ_2	1	-1	1	-1
Γ_3	1	-1	-1	1
Γ_4	1	1	-1	-1
Γ_5	$-\mathbb{1}$	$-\mathrm{i}\sigma_y$	σ_x	$-\sigma_z$

M

	$\{2\|(0,0)\}$	$\{4^+\|(0,0)\}$	$\{m_{01}\|(1/2,1/2)\}$	$\{m_{1\bar{1}}\|(1/2,1/2)\}$
M_1	-1	i	$-$i	-1
M_2	-1	$-$i	$-$i	1
M_3	-1	$-$i	i	-1
M_4	-1	i	i	1
M_5	$\mathbb{1}$	σ_x	$\mathrm{i}\sigma_y$	σ_z

X

	$\{2\|(0,0)\}$	$\{m_{01}\|(1/2,1/2)\}$	$\{m_{1\bar{1}}\|(1/2,1/2)\}$
X_1	σ_x	σ_y	$-\mathrm{i}\sigma_z$

$\overline{\Gamma X}$

	$\{m_{10}\|(1/2,1/2)\}$
DT_1	$\exp(\pi u\mathrm{i})$
DT_2	$\exp(\pi[1+u]\mathrm{i})$

$\overline{\Gamma M}$

	$\{m_{1\bar{1}}\|(1/2,1/2)\}$
SM_1	$\exp(2\pi u\mathrm{i})$
SM_2	$\exp(\pi[1+2u]\mathrm{i})$

$\overline{XM}$

	$\{m_{01}\|(1/2,1/2)\}$
Y_1	$\exp(\pi u\mathrm{i})$
Y_2	$\exp(\pi[1+u]\mathrm{i})$

TABLE S11: The relevant irreducible representations of $p4gm$ at the high-symmetry points $\Gamma = (0,0)$, $M = (1/2,1/2)$, and $X = (0,1/2)$ as well as along the lines $\overline{\Gamma X} = (0,u)$, $\overline{\Gamma M} = (u,u)$, and $\overline{XM} = (u,1/2)$.

The fragile roots in $p4gm$ [S4] are given in Tab. S12; the elementary band representations can be retrieved from the Bilbao server.

#	root	# of bands	type
1	$2\Gamma_2 + 2\Gamma_3 + M_1M_3 + M_2M_4 + 2X_1$	4	conjugate pairs & Chern
2	$2\Gamma_1 + 2\Gamma_4 + M_1M_3 + M_2M_4 + 2X_1$	4	conjugate pairs & Chern
3	$\Gamma_1 + \Gamma_4 + \Gamma_5 + M_1M_3 + M_2M_4 + 2X_1$	4	conjugate pairs & 2D irrep
4	$\Gamma_2 + \Gamma_3 + \Gamma_5 + M_1M_3 + M_2M_4 + 2X_1$	4	conjugate pairs & 2D irrep

TABLE S12: Fragile roots and their types in wallpaper group $p4gm$.

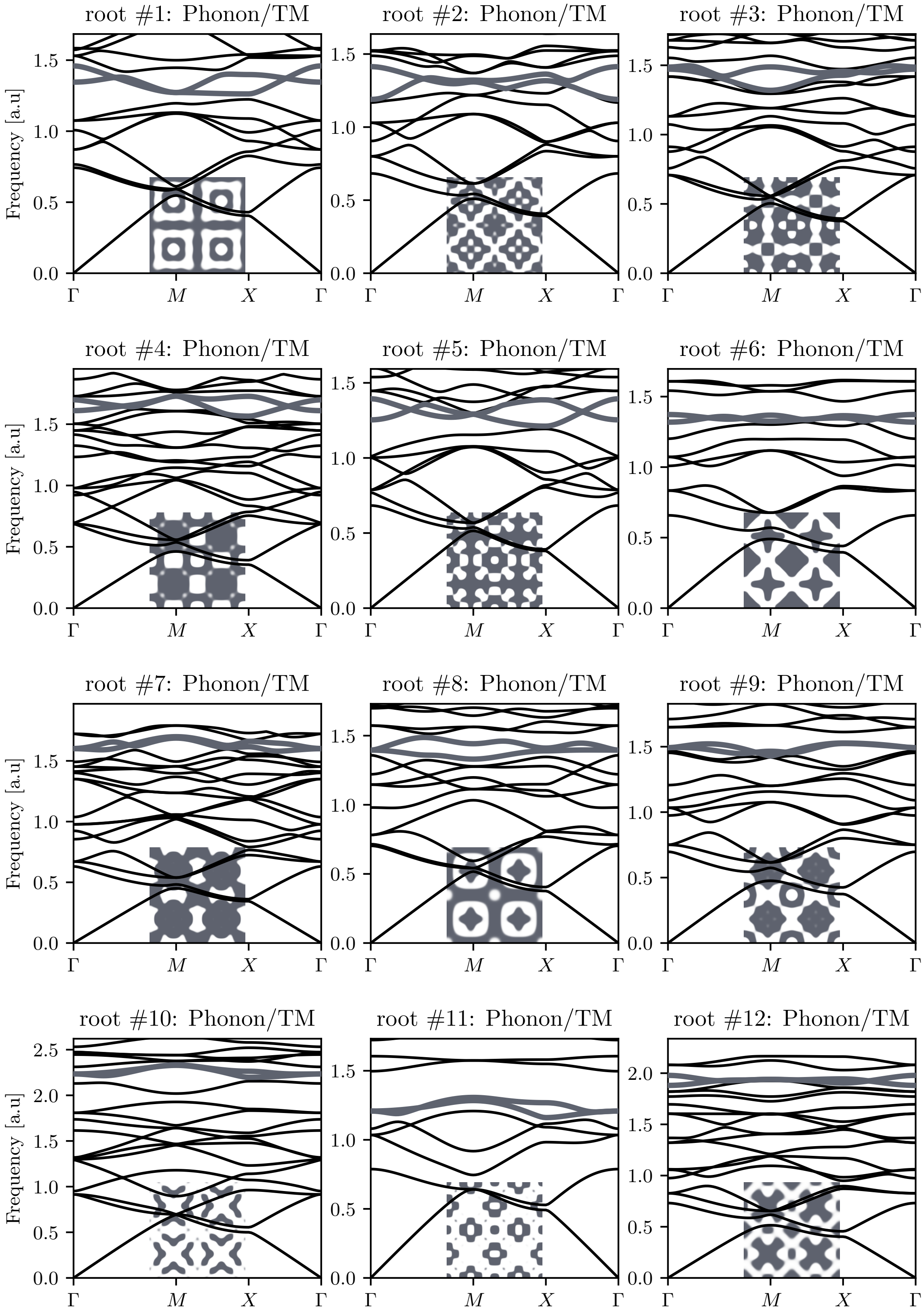

FIG. S19: One sample for each of the roots in $p4mm$ for phonons and TM photons.

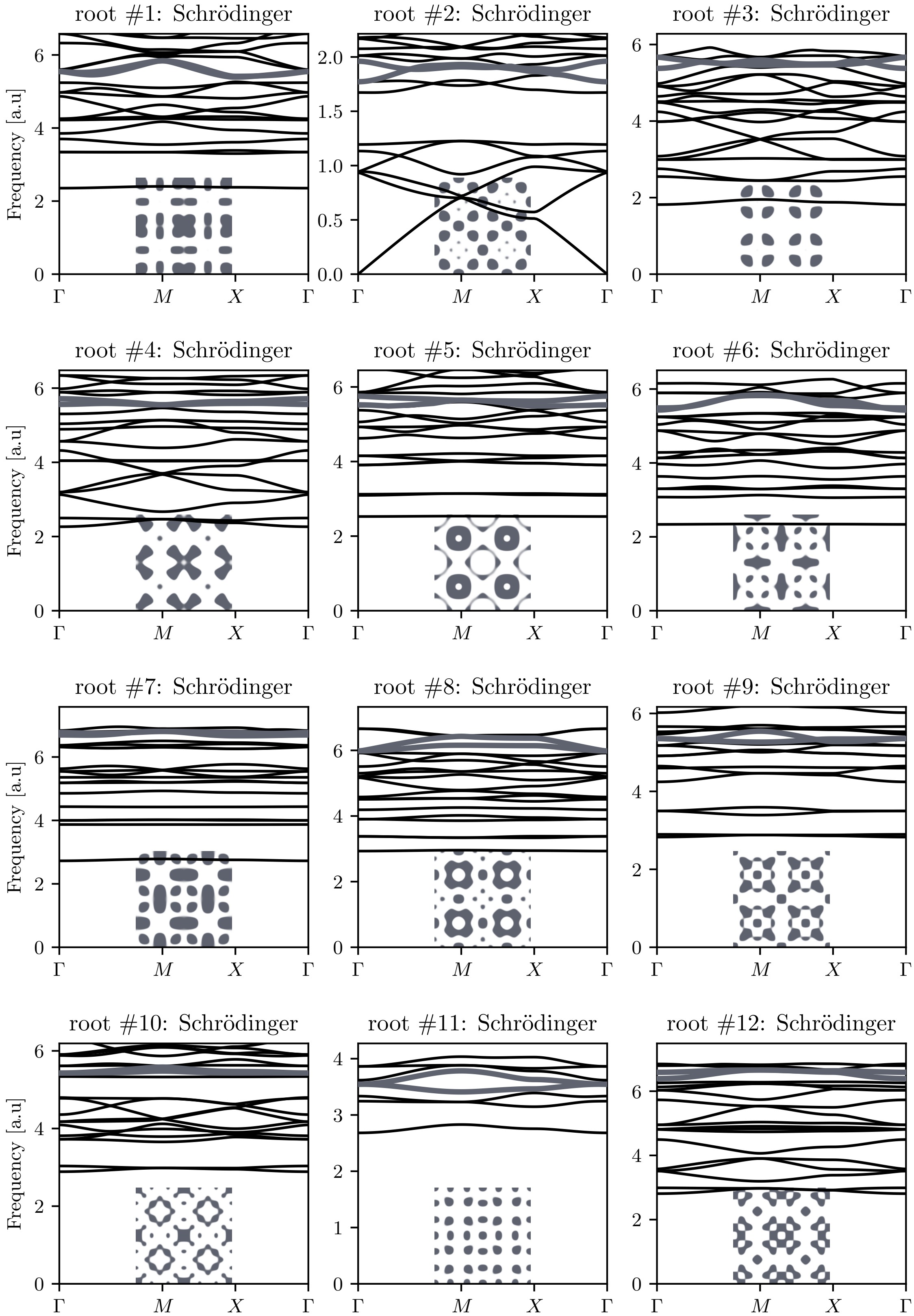

FIG. S20: One sample for each of the roots in *p4mm* for systems described by the Schrödinger equation.

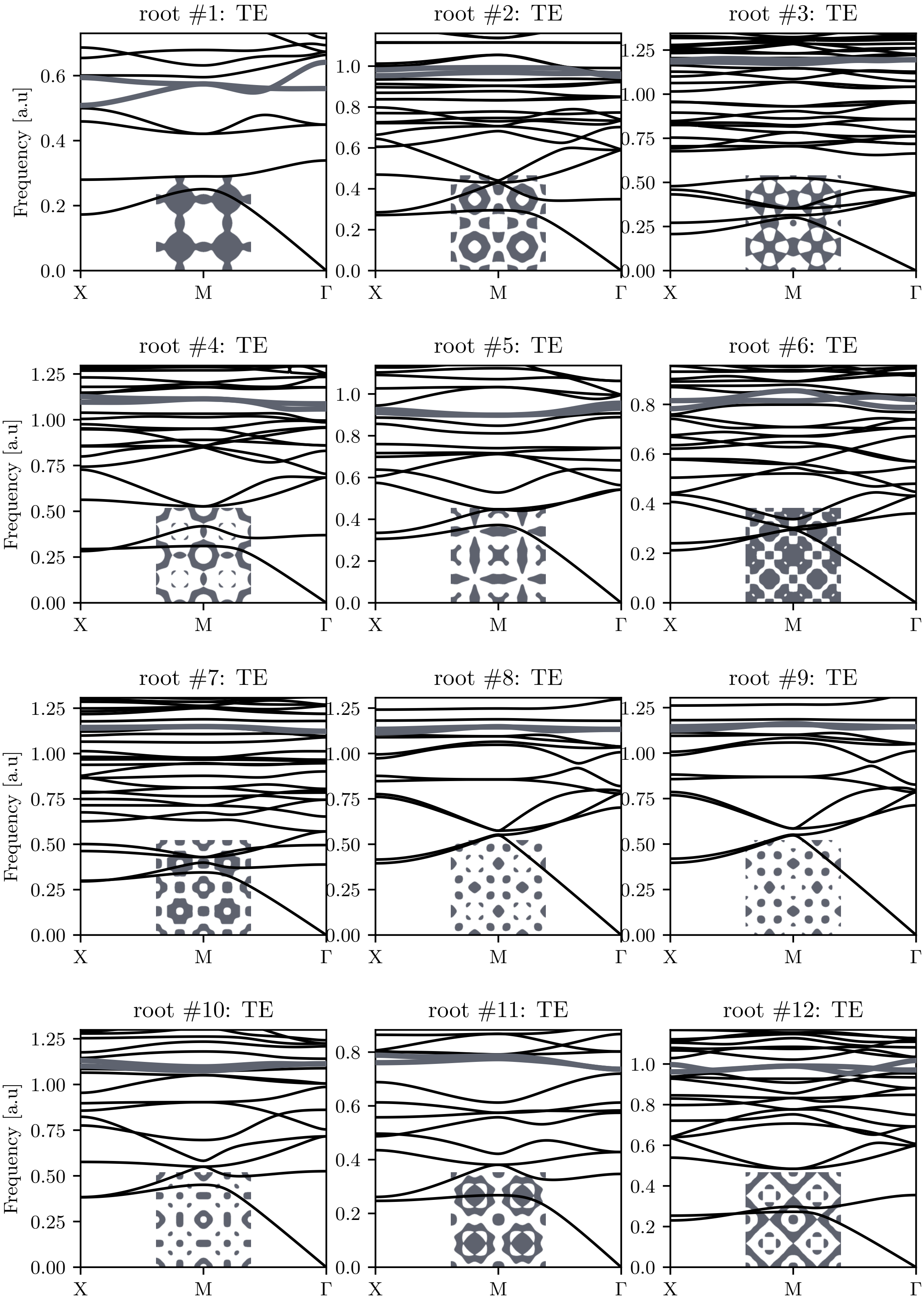

FIG. S21: One sample for each of the roots in *p4mm* for TE photons.

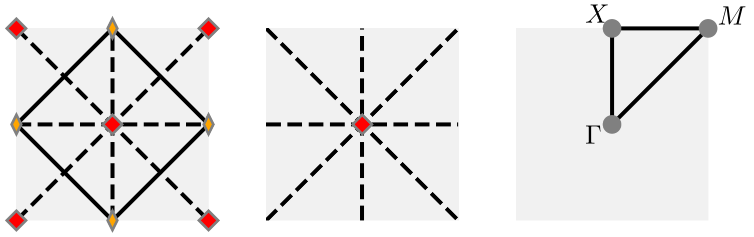

FIG. S22: Left: Unit cell with the full symmetry of *p4gm*. Middle: General positions of *p4gm*. Right: Brillouin zone with the high-symmetry points and lines indicated.

2. *Bundling strategy*

We analyze the structure of the roots in the wallpaper group *p4gm* shown in Tab. S12. As we will see, we need to invoke the vanishing Chern number argument to argue for the bundling of bands into fragile roots. We start with root #1

$$2\Gamma_2 + 2\Gamma_3 + M_1M_3 + M_2M_4 + 2X_1. \tag{S32}$$

Let us see who we can link up with whom. For this, we consult the tables with the irreducible representations at Γ, M, X, and along the line $\overline{\Gamma X} = (u, u)$ in Tab. S11.

Let us start with the conjugate pair M_1M_3 which both are odd under $\{m_{1\bar{1}}|(1/2, 1/2)\}$ and, accordingly, are compatible with SM_1, which in turn forces us to connect M_1M_3 to $2\Gamma_3$. In the other pair M_2M_4, both transform evenly under $\{m_{1\bar{1}}|(1/2, 1/2)\}$ and are therefore via SM_2 connected to $2\Gamma_2$. Along $\overline{XM}$, the irreps Y_1 and Y_2 turn the eigenvalues $\pm i$ of $\{m_{01}|(1/2, 1/2)\}$ of M_2M_4 and M_1M_3 into ± 1 of X_1, cf. Tab. S11. Moreover, the non-symmorphic $\{m_{01}|(1/2, 1/2)\}$ together with the time-reversal symmetry $T = K$, with K denoting complex conjugation, gives rise to effective Kramers pair. In other words, we find ourselves in the typical situation of band-stickings along the zone boundary.

Hence, from compatibility relations alone, we would bundle

$$2\Gamma_2 + 2\Gamma_3 + M_1M_3 + M_2M_4 + 2X_1 = (2\Gamma_2 + M_2M_4 + X_1) \oplus (2\Gamma_3 + M_1M_3 + X_1) \tag{S33}$$

Using formula (S23) we conclude that we have

$$(2\Gamma_2 + M_2M_4 + X_1): \quad \mathrm{i}^C = (-1)^2 \times -\mathrm{i} \times \mathrm{i} \times -1 \times 1 = -1 \quad \rightarrow \quad C = 2 + 4n \ \text{ with } \ n \in \mathbb{Z} \tag{S34}$$

$$(2\Gamma_3 + M_1M_2 + X_1): \quad \mathrm{i}^C = (-1)^2 \times \mathrm{i} \times -\mathrm{i} \times -1 \times 1 = -1 \quad \rightarrow \quad C = 2 + 4n \ \text{ with } \ n \in \mathbb{Z} \tag{S35}$$

From this we read that (i) for one of the two $n = 0$ and for the other $n = -1$, such that by having (ii) a gap-closing between the two sets of bands, we deal with a *bundle of four bands* with zero Chern number.

We now consider the next root #2

$$2\Gamma_1 + 2\Gamma_4 + M_1M_3 + M_2M_4 + 2X_1 \tag{S36}$$

The same argument as above leads us to the conclusion that we can write

$$2\Gamma_1 + 2\Gamma_4 + M_1M_3 + M_2M_4 + 2X_1 = (2\Gamma_4 + M_2M_4 + X_1) \oplus (2\Gamma_1 + M_1M_3 + X_1) \tag{S37}$$

And again, through the argument that the Chern number has to be zero, we conclude that we have a bundle of four bands with band touchings off high-symmetry lines or points.

For the two remaining roots of this group

$$\Gamma_1 + \Gamma_4 + \Gamma_5 + M_1M_3 + M_2M_4 + 2X_1 \tag{S38}$$

$$\Gamma_2 + \Gamma_3 + \Gamma_5 + M_1M_3 + M_2M_4 + 2X_1 \tag{S39}$$

we see that of the two $\{m_{1\bar{1}}|(1/2, 1/2)\}$-even (odd) lines emerging from M_2M_4 (M_1M_3), which along $\overline{\Gamma M}$ turn $\{m_{1\bar{1}}|(1/2, 1/2)\}$-odd (even), one has to be absorbed by $\Gamma_{4/2}$ ($\Gamma_{1/3}$) and one by Γ_5, respectively. In other words: The four bands are linked together at high-symmetry points as in Fig. S23.

Finally, the types of the roots of *p4gm* are indicated in the last column of Tab. S12.

3. *Examples*

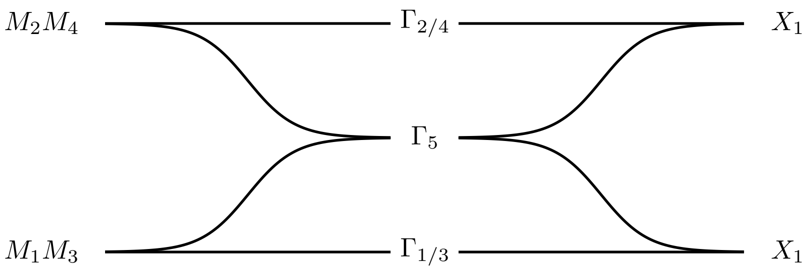

FIG. S23: Linkage of bands for the fragile roots #3 and #4 of $p4gm$.

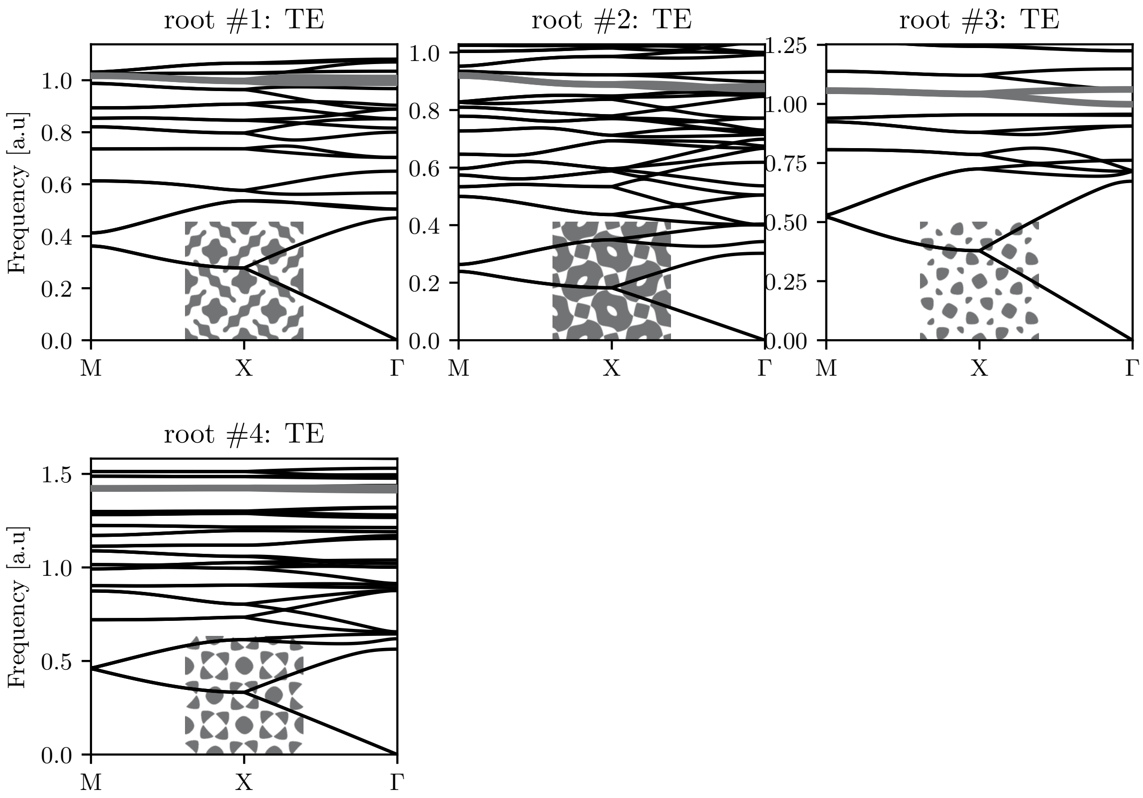

FIG. S24: One sample for each of the roots in $p4gm$ for TE photons.

H. $p3$

1. Basic group properties

The group $p3$ describes a hexagonal lattice with lattice vectors $\boldsymbol{a}_1 = (1, 0)$ and $\boldsymbol{a}_2 = (-\sqrt{3}/2, 1/2)$. The corresponding space group is $P3$, (#143) constrained to the x-y plane. The group $p3$ contains the following group elements [retrieve from Bilbao server]

$$\{1|n_1\boldsymbol{a}_1 + n_2\boldsymbol{a}_2\}, \quad \text{with} \quad n_1, n_2 \in \mathbb{Z} \tag{S40}$$

$$\{3^+|(0,0)\},\ \{3^-|(0,0)\}. \tag{S41}$$

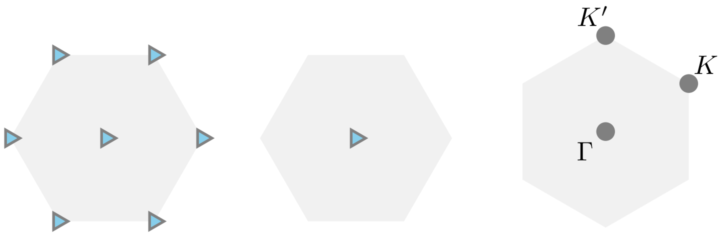

FIG. S25: Left: Unit cell with the full symmetry of $p3$. Middle: General positions of $p3$. Right: Brillouin zone with the high-symmetry points and lines indicated.

The relevant members of the little groups of the high-symmetry points and lines and their irreps are given in Tab. S13. The full set can be obtained from the Bilbao Server.

$\Omega = \Gamma,\, K$

	$\{3^+\|(0,0)\}$
Ω_1	1
Ω_2	z^2
Ω_3	$\bar{z}^2$

K'

	$\{3^+\|(0,0)\}$
KA_1	1
KA_2	$\bar{z}^2$
KA_3	z^2

TABLE S13: The relevant irreducible representations of $p3$ at the high-symmetry points $\Gamma = (0,0)$, $K = (1/3, 1/3)$, and $K' = (-1/3, -1/3)$. Note that $z = \exp(i\pi/3)$.

The fragile roots in $p3$ [S4] are given in Tab. S14; the elementary band representations can be retrieved from the Bilbao server.

#	root	# of bands	type
1	$\Gamma_2\Gamma_3 + 2K_2 + 2KA_2 + 2M_1$	2	conjugate pairs
2	$\Gamma_2\Gamma_3 + 2K_1 + 2KA_1 + 2M_1$	2	conjugate pairs
3	$\Gamma_2\Gamma_3 + 2K_3 + 2KA_3 + 2M_1$	2	conjugate pairs

TABLE S14: Fragile roots and their types in wallpaper group $p3$.

2. Bundling strategy

In all fragile roots of Tab. S13, only the conjugate pairs $\Gamma_2\Gamma_3$ appear at the Γ-point. Hence, a bundling according to these is sufficient as shown in the last column of Tab. S14.

3. Examples

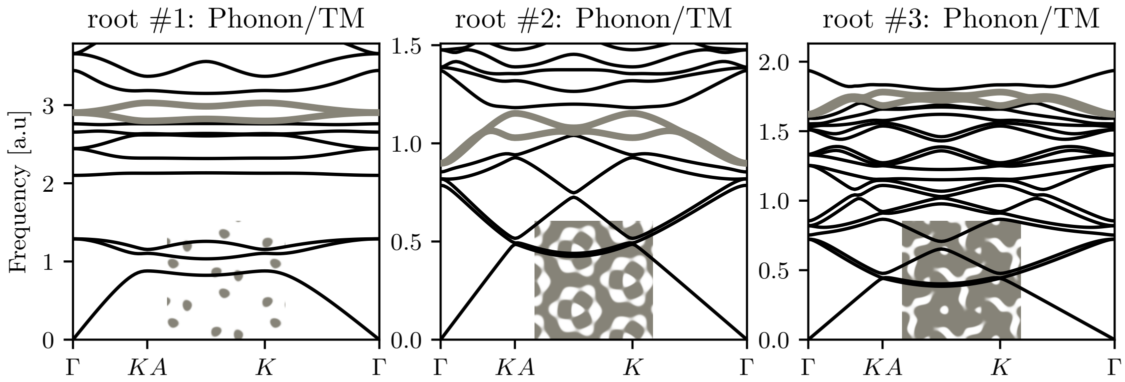

FIG. S26: One sample for each of the roots in $p3$ for phonons and TM photons.

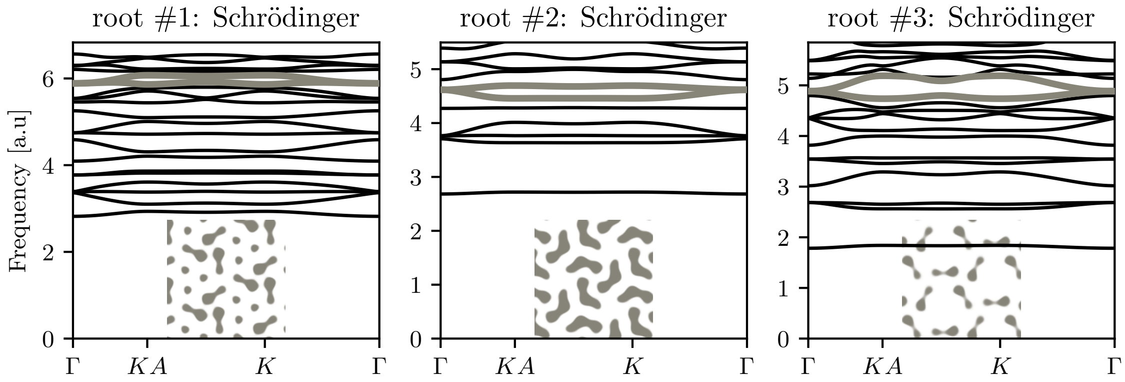

FIG. S27: One sample for each of the roots in $p3$ for systems described by the Schrödinger equation.

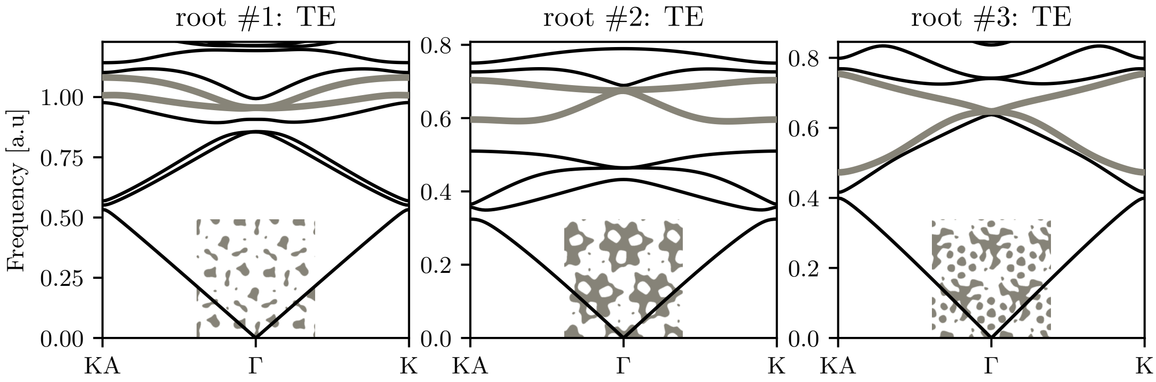

FIG. S28: One sample for each of the roots in $p3$ for TE photons.

I. $p3m1$

1. *Basic group properties*

The group $p3m1$ describes a hexagonal lattice with lattice vectors $\boldsymbol{a}_1 = (1, 0)$ and $\boldsymbol{a}_2 = (-\sqrt{3}/2, 1/2)$. The corresponding space group is $P3m1$, (#156) constrained to the x-y plane. The group $p3m1$ contains the following

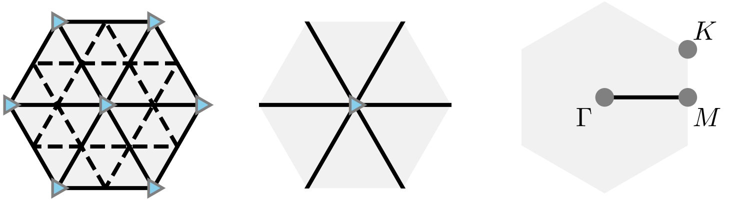

FIG. S29: Left: Unit cell with the full symmetry of $p3m1$. Middle: General positions of $p3m1$. Right: Brillouin zone with the high-symmetry points and lines indicated.

group elements [retrieve from Bilbao server]

$$\{1|n_1\boldsymbol{a}_1 + n_2\boldsymbol{a}_2\}, \quad \text{with} \quad n_1, n_2 \in \mathbb{Z} \tag{S42}$$

$$\{3^+|(0,0)\},\ \{3^-|(0,0)\}, \tag{S43}$$

$$\{m_{11}|(0,0)\},\ \{m_{10}|(0,0)\},\ \{m_{01}|(0,0)\}. \tag{S44}$$

The relevant members of the little groups of the high-symmetry points and lines and their irreps are given in Tab. S15. The full set can be obtained from the Bilbao Server.

Γ

	$\{3^+\|(0,0)\}$	$\{m_{11}\|(0,0)\}$	$\{m_{10}\|(0,0)\}$	$\{m_{01}\|(0,0)\}$
Γ_1	1	1	1	1
Γ_2	1	-1	-1	-1
Γ_3	$\begin{pmatrix} z^2 & 0 \\ 0 & \bar{z}^2 \end{pmatrix}$	σ_x	$\begin{pmatrix} 0 & \bar{z}^2 \\ z^2 & 0 \end{pmatrix}$	$\begin{pmatrix} 0 & z^2 \\ \bar{z}^2 & 0 \end{pmatrix}$

K

	$\{3^+\|(0,0)\}$
K_1	1
K_2	z^2
K_3	$\bar{z}^2$

M

	$\{m_{01}\|(0,0)\}$
M_1	1
M_2	-1

$\overline{\Gamma M}$

	$\{m_{01}\|(0,0)\}$
SM_1	1
SM_2	-1

TABLE S15: The relevant irreducible representations of $p3m1$ at the high-symmetry points $\Gamma = (0,0)$, $M = (1/2, 0)$, and $K = (1/3, 1/3)$. Note that $z = \exp(\mathrm{i}\pi/3)$.

The fragile roots in $p3m1$ [S4] are given in Tab. S16; the elementary band representations can be retrieved from the Bilbao server.

#	root	# of bands	type
1	$\Gamma_3 + 2K_2 + M_1 + M_2$	2	2D irrep
2	$\Gamma_3 + 2K_1 + M_1 + M_2$	2	2D irrep
3	$\Gamma_3 + 2K_3 + M_1 + M_2$	2	2D irrep

TABLE S16: Fragile roots and their types in wallpaper group $p3m1$.

2. *Bundling strategy*

In all three roots, the two-dimensional irrep Γ_3 is appearing. Hence, the bands are glued together at the origin of the Brillouin zone as indicated in the last column of Tab. S16

3. *Examples*

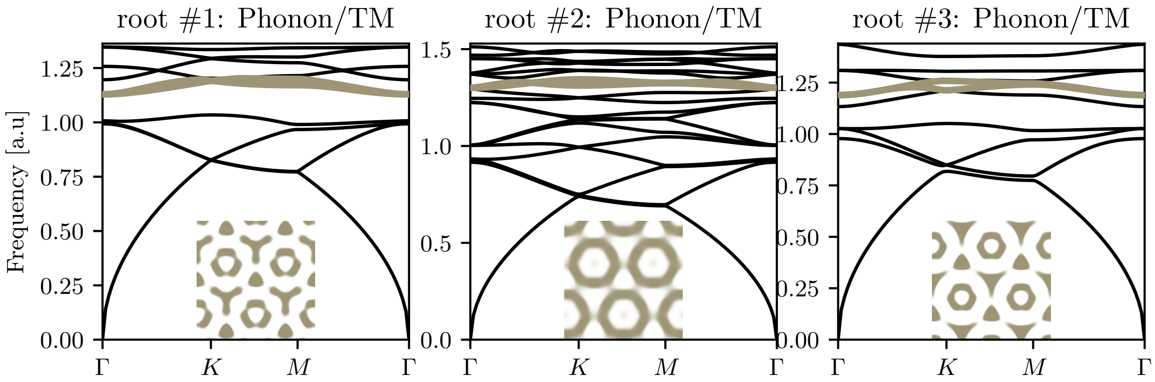

FIG. S30: One sample for each of the roots in $p3m1$ for phonons and TM photons.

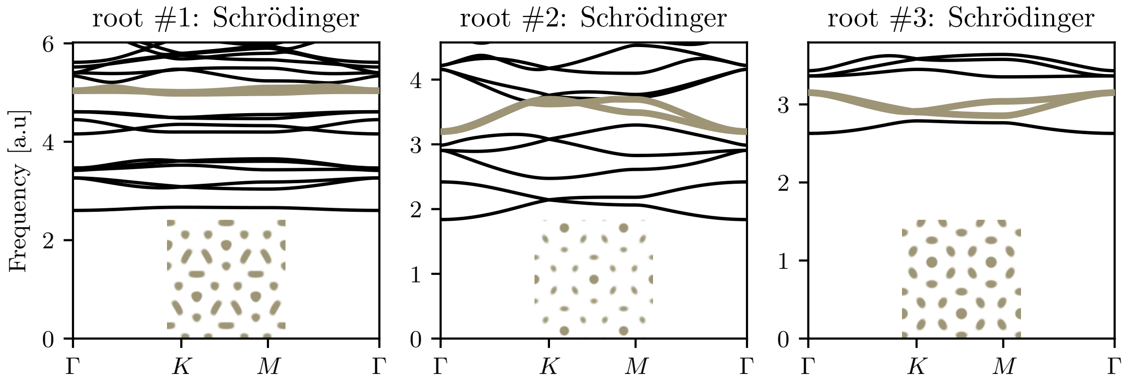

FIG. S31: One sample for each of the roots in $p3m1$ for systems described by the Schrödinger equation.

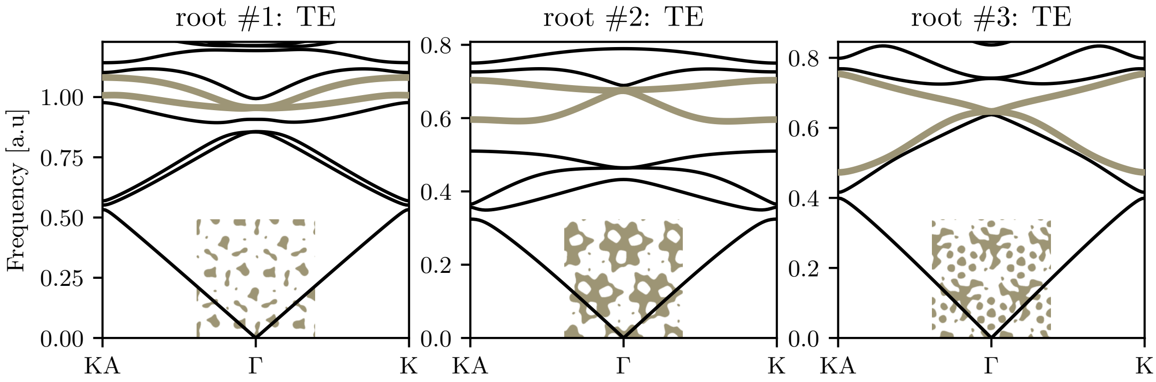

FIG. S32: One sample for each of the roots in $p3m1$ for TE photons.

J. $p31m$

1. *Basic group properties*

The group $p31m$ describes a hexagonal lattice with lattice vectors $\boldsymbol{a}_1 = (1, 0)$ and $\boldsymbol{a}_2 = (-\sqrt{3}/2, 1/2)$. The corresponding space group is $P31m$, (#157) constrained to the x-y plane. The group $p31m$ contains the following

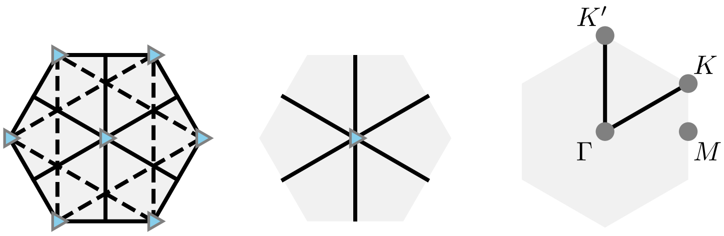

FIG. S33: Left: Unit cell with the full symmetry of $p31m$. Middle: General positions of $p31m$. Right: Brillouin zone with the high-symmetry points and lines indicated.

group elements [retrieve from Bilbao server]

$$\{1|n_1\boldsymbol{a}_1 + n_2\boldsymbol{a}_2\}, \quad \text{with} \quad n_1, n_2 \in \mathbb{Z} \tag{S45}$$

$$\{3^+|(0,0)\},\ \{3^-|(0,0)\}, \tag{S46}$$

$$\{m_{1\bar{1}}|(0,0)\},\ \{m_{12}|(0,0)\},\ \{m_{21}|(0,0)\}. \tag{S47}$$

The relevant members of the little groups of the high-symmetry points and lines and their irreps are given in Tab. S17. The full set can be obtained from the Bilbao Server.

$\Omega = \Gamma,\, K$

	$\{3^+\|(0,0)\}$	$\{m_{1\bar{1}}\|(0,0)\}$	$\{m_{12}\|(0,0)\}$	$\{m_{21}\|(0,0)\}$
Ω_1	1	1	1	1
Ω_2	1	-1	-1	-1
Ω_3	$\begin{pmatrix} z^2 & 0 \\ 0 & \bar{z}^2 \end{pmatrix}$	σ_x	$\begin{pmatrix} 0 & \bar{z}^2 \\ z^2 & 0 \end{pmatrix}$	$\begin{pmatrix} 0 & z^2 \\ \bar{z}^2 & 0 \end{pmatrix}$

K'

	$\{3^+\|(0,0)\}$	$\{m_{1\bar{1}}\|(0,0)\}$	$\{m_{12}\|(0,0)\}$	$\{m_{21}\|(0,0)\}$
KA_1	1	1	1	1
KA_2	1	-1	-1	-1
KA_3	$\begin{pmatrix} \bar{z}^2 & 0 \\ 0 & z^2 \end{pmatrix}$	σ_x	$\begin{pmatrix} 0 & z^2 \\ \bar{z}^2 & 0 \end{pmatrix}$	$\begin{pmatrix} 0 & \bar{z}^2 \\ z^2 & 0 \end{pmatrix}$

M

	$\{m_{21}\|(0,0)\}$
M_1	1
M_2	-1

$\overline{K'\Gamma K}$

	$\{m_{1\bar{1}}\|(0,0)\}$
LD_1	1
LD_2	-1

TABLE S17: The relevant irreducible representations of $p31m$ at the high-symmetry points $\Gamma = (0,0)$, $M = (1/2, 0)$, $K = (1/3, 1/3)$ and $K' = (-1/3, -1/3)$ as wall as on the line $\overline{K'\Gamma K}$. Note that $z = \exp(\mathrm{i}\pi/3)$.

The fragile root in $p31m$ [S4] is given in Tab. S18; the elementary band representations can be retrieved from the Bilbao server.

#	root	# of bands	type
1	$\Gamma_3 + K_1 + K_2 + KA_1 + KA_2 + M_1 + M_2$	2	2D irrep

TABLE S18: The fragile roots and its type in wallpaper group $p31m$.

2. *Bundling strategy*

The two-dimensional irrep Γ_3 is appearing in the only fragile root of $p31m$. Therefore, bundling can be trivially achieved using this double degeneracy as shown in the last column of Tab. S18.

3. *Examples*

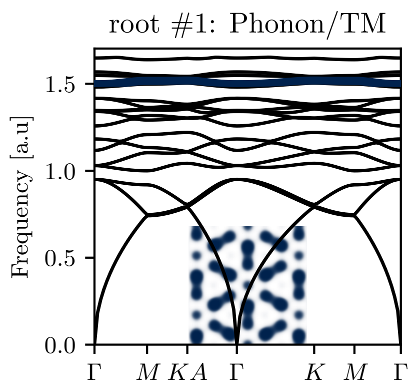

FIG. S34: One sample for each of the roots in $p31m$ for phonons and TM photons.

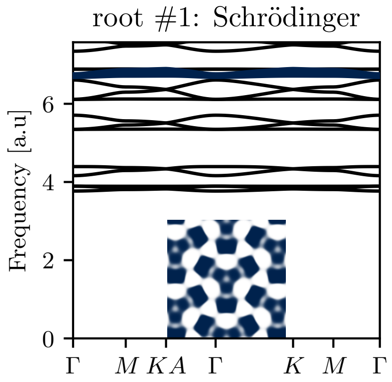

FIG. S35: One sample for each of the roots in $p31m$ for systems described by the Schrödinger equation.

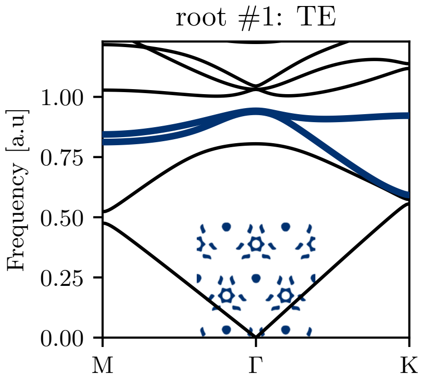

FIG. S36: One sample for each of the roots in $p31m$ for TE photons.

K. $p6$

1. *Basic group properties*

The group $p6$ describes a hexagonal lattice with lattice vectors $\boldsymbol{a}_1 = (1, 0)$ and $\boldsymbol{a}_2 = (-\sqrt{3}/2, 1/2)$. The corresponding space group is $P6$, (#168) constrained to the x-y plane. The group $p6mm$ contains the following group elements [retrieve from Bilbao server]

$$\{1|n_1\boldsymbol{a}_1 + n_2\boldsymbol{a}_2\}, \quad \text{with} \quad n_1, n_2 \in \mathbb{Z} \tag{S48}$$

$$\{2|(0,0)\},\ \{3^+|(0,0)\},\ \{3^-|(0,0)\},\ \{6^+|(0,0)\},\ \{6^-|(0,0)\}. \tag{S49}$$

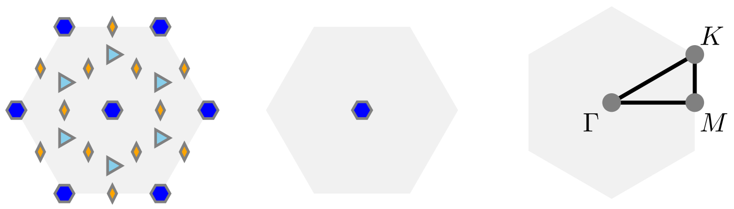

FIG. S37: Left: Unit cell with the full symmetry of $p6$. Middle: General positions of $p6$. Right: Brillouin zone with the high-symmetry points and lines indicated.

The relevant members of the little groups of the high-symmetry points and lines and their irreps are given in Tab. S21. The full set can be obtained from the Bilbao Server.

Γ

	$\{6^+\|(0,0)\}$
Γ_1	1
Γ_2	-1
Γ_3	$\bar{z}^2$
Γ_4	z
Γ_5	z^2
Γ_6	$\bar{z}$

K

	$\{3^+\|(0,0)\}$
K_1	1
K_2	z^2
K_3	$\bar{z}^2$

M

	$\{2\|(0,0)\}$
M_1	1
M_2	-1

TABLE S19: The relevant irreducible representations of $p6$ at the high-symmetry points $\Gamma = (0,0)$, $M = (1/2, 0)$, and $K = (1/3, 1/3)$. Note that $z = \exp(\mathrm{i}\pi/3)$.

The fragile roots in $p6$ [S4] are given in Tab. S20; the elementary band representations can be retrieved from the Bilbao server.

#	root	# of bands	type
1	$2\Gamma_2 + 2K_1 + 2M_1$	2	Chern
2	$2\Gamma_2 + K_2K_3 + 2M_1$	2	conjugate pairs
3	$2\Gamma_2 + K_2K_3 + 2M_2$	2	conjugate pairs
4	$2\Gamma_1 + 2K_1 + 2M_2$	2	Chern
5	$2\Gamma_1 + K_2K_3 + 2M_2$	2	conjugate pairs
6	$2\Gamma_1 + K_2K_3 + 2M_1$	2	conjugate pairs
7	$\Gamma_4\Gamma_6 + 2K_1 + 2M_1$	2	conjugate pairs
8	$\Gamma_3\Gamma_5 + 2K_1 + 2M_1$	2	conjugate pairs
9	$\Gamma_3\Gamma_5 + K_2K_3 + 2M_2$	2	conjugate pairs
10	$\Gamma_4\Gamma_6 + K_2K_3 + 2M_1$	2	conjugate pairs
11	$\Gamma_4\Gamma_6 + 2K_1 + 2M_2$	2	conjugate pairs
12	$\Gamma_3\Gamma_5 + 2K_1 + 2M_2$	2	conjugate pairs

TABLE S20: Fragile roots and their types in wallpaper group $p6$.

2. Bundling strategies

Let us start with the first two roots #1 and #4, where only one dimensional irreps with real characters are involved. Moreover, no mirrors help us in further constraining the bundling. This means, in principle, we could write for #1

$$(\Gamma_2 + K_1 + M_1) \oplus (\Gamma_2 + K_1 + M_1). \tag{S50}$$

We now invoke the usual Chern argument by writing [S6]

$$e^{\mathrm{i}\pi C/3} = \prod_{i \,\in\, \mathrm{occ.}} (-1)^F \eta_i(\Gamma)\vartheta_i(K)\zeta_i(M), \tag{S51}$$

where $F = 2S$, with S the total spin of the particles, η, ϑ, and ζ are the eigenvalues of $\{6^+|(0,0)\}$ and $\{3^+|(0,0)\}$, and $\{2|(0,0)\}$ respectively. In the following we set $F = 0$. For the bands above, this means, the Chern number C of these bands is given by

$$C = 3 + 6n \quad \text{with} \quad n \in \mathbb{Z}. \tag{S52}$$

The only way this is compatible with time reversal symmetry is by having a band touching between the sets of bands. The same holds for root #4.

For all remaining roots, there is either at K or at Γ a pair of conjugate irreps which glues the bands together. Finally, the types of the roots of $p6$ are indicated in the last column of Tab. S20.

3. Examples

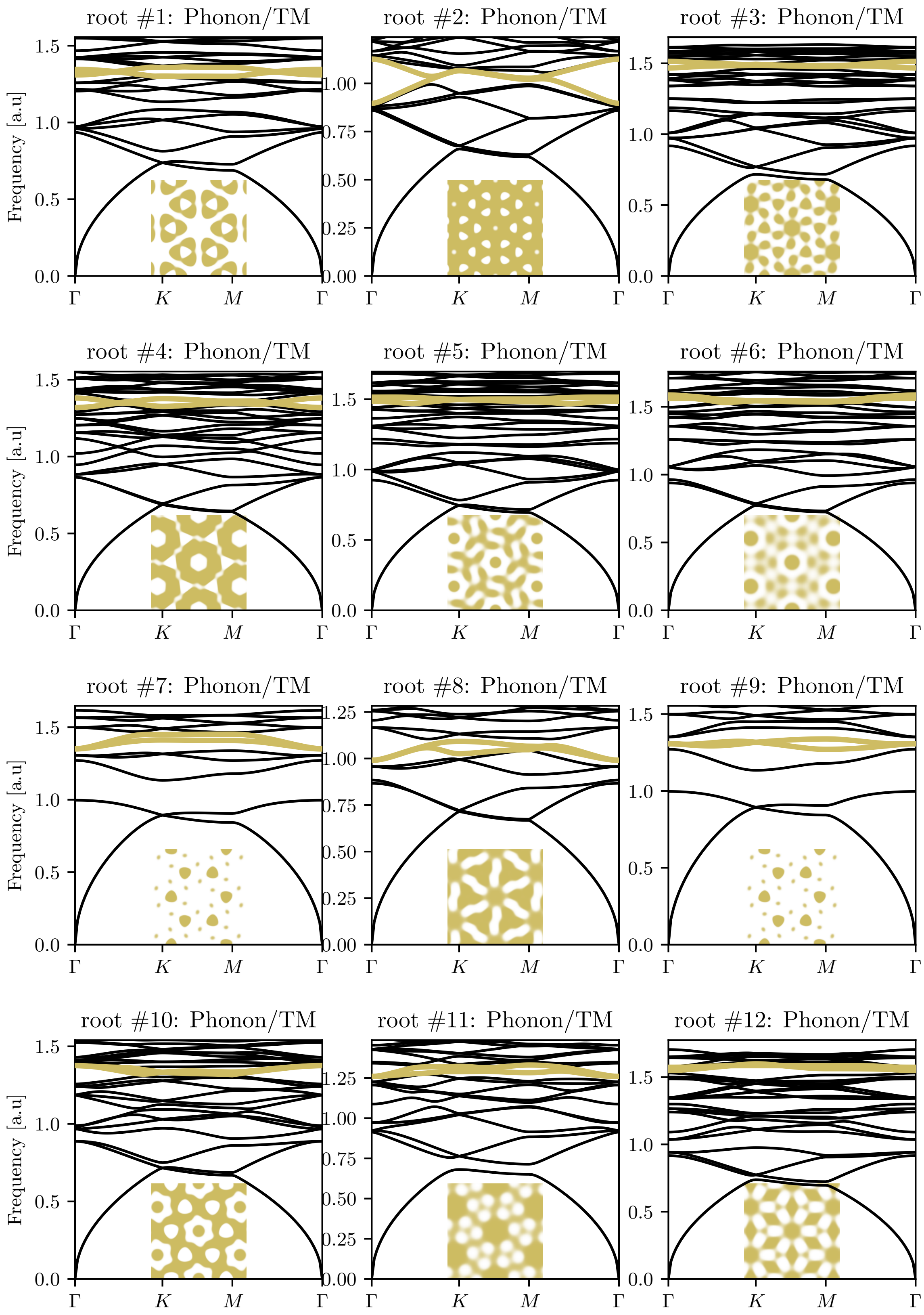

FIG. S38: One sample for each of the roots in $p6$ for phonons and TM photons.

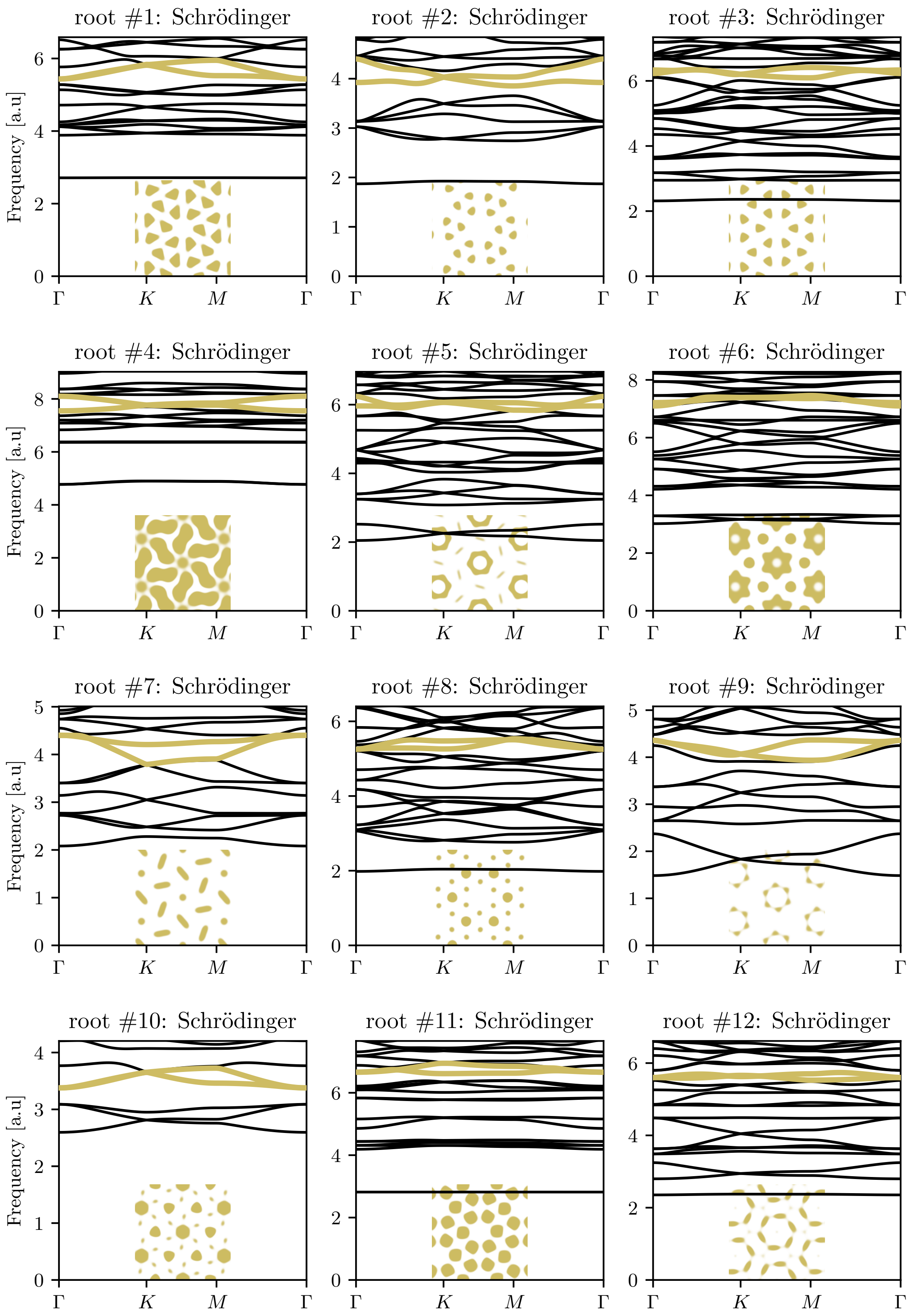

FIG. S39: One sample for each of the roots in $p6$ for systems described by the Schrödinger equation.

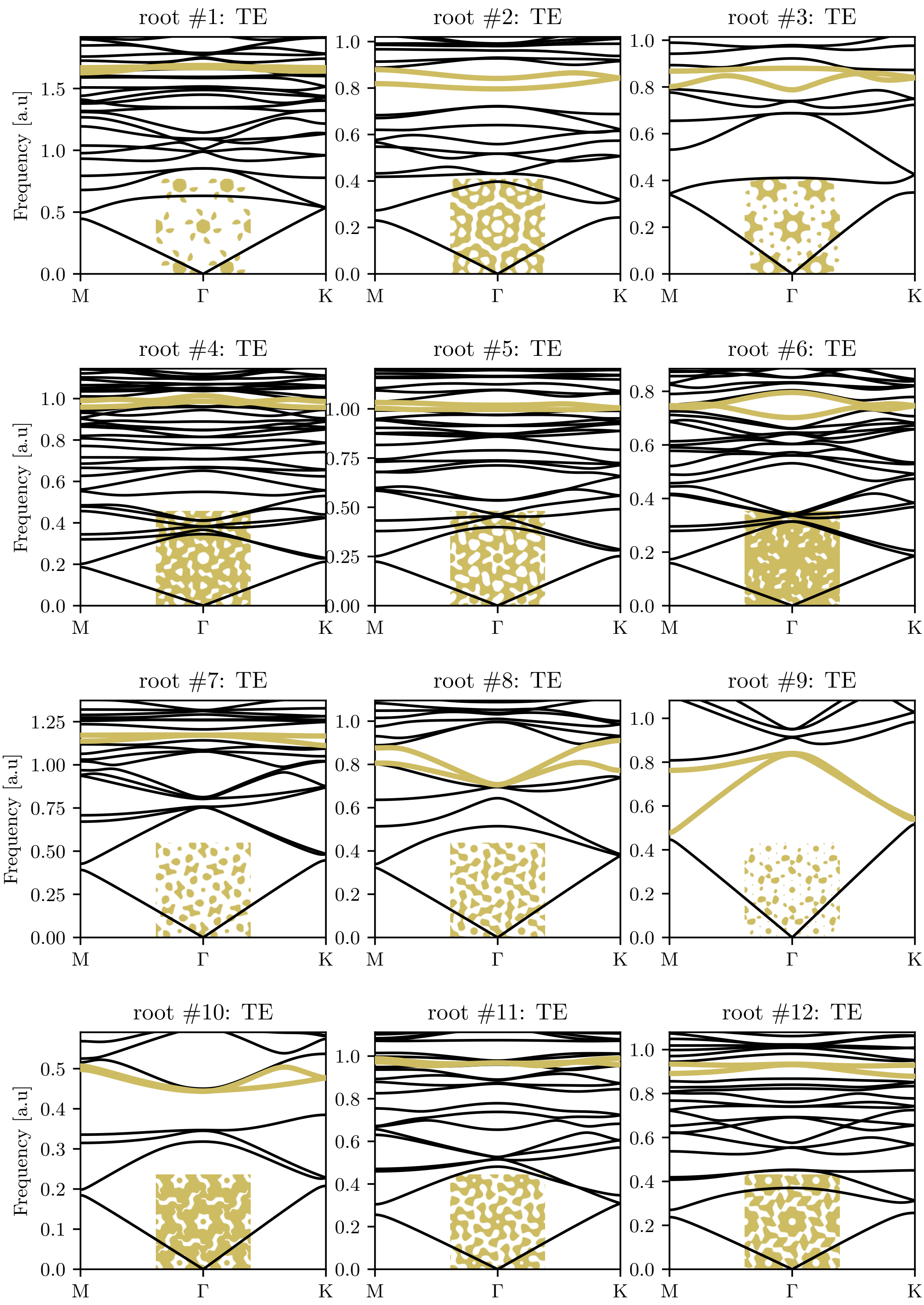

FIG. S40: One sample for each of the roots in $p6$ for TE photons.

L. *p*6*mm*

1. *Basic group properties*

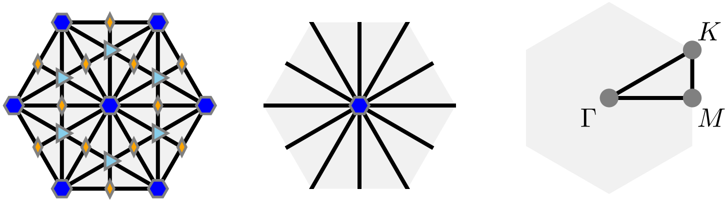

FIG. S41: Left: Unit cell with the full symmetry of $p6mm$. (There are additional glide planes parallel to the mirror planes, always halfway in between the mirror planes. They are not shown for simplicity). Middle: General positions of $p6mm$. Right: Brillouin zone with the high-symmetry points and lines indicated.

The group $p6mm$ describes a hexagonal lattice with lattice vectors $\boldsymbol{a}_1 = (1,0)$ and $\boldsymbol{a}_2 = (-\sqrt{3}/2, 1/2)$. The corresponding space group is $P6mm$, (#183) constrained to the x-y plane. The group $p6mm$ contains the following group elements [retrieve from Bilbao server]

$$\{1|n_1\boldsymbol{a}_1 + n_2\boldsymbol{a}_2\}, \quad \text{with} \quad n_1, n_2 \in \mathbb{Z} \tag{S53}$$

$$\{2|(0,0)\},\ \{3^+|(0,0)\},\ \{3^-|(0,0)\},\ \{6^+|(0,0)\},\ \{6^-|(0,0)\} \tag{S54}$$

$$\{m_{11}|(0,0)\},\ \{m_{10}|(0,0)\},\ \{m_{01}|(0,0)\},\ \{m_{1\bar{1}}|(0,0)\},\ \{m_{12}|(0,0)\},\ \{m_{21}|(0,0)\}. \tag{S55}$$

The relevant members of the little groups of the high-symmetry points and lines and their irreps are given in Tab. S21. The full set can be obtained from the Bilbao Server.

Γ

	$\{6^+\|(0,0)\}$	$\{m_{01}\|(0,0)\}$	$\{m_{1\bar{1}}\|(0,0)\}$
Γ_1	1	1	1
Γ_2	1	−1	−1
Γ_3	−1	−1	1
Γ_4	−1	1	−1
Γ_5	$\begin{pmatrix}\bar{z}^2 & 0\\ 0 & z^2\end{pmatrix}$	$\begin{pmatrix}0 & \bar{z}^2\\ z^2 & 0\end{pmatrix}$	σ_x
Γ_6	$\begin{pmatrix}z & 0\\ 0 & \bar{z}\end{pmatrix}$	$\begin{pmatrix}0 & z^2\\ \bar{z}^2 & 0\end{pmatrix}$	$-\sigma_x$

K

	$\{3^+\|(0,0)\}$	$\{m_{1\bar{1}}\|(0,0)\}$	$\{m_{21}\|(0,0)\}$
K_1	1	1	1
K_2	1	−1	−1
K_3	$\begin{pmatrix}\bar{z}^2 & 0\\ 0 & z^2\end{pmatrix}$	σ_x	$\begin{pmatrix}0 & z^2\\ \bar{z}^2 & 0\end{pmatrix}$

M

	$\{2\|(0,0)\}$	$\{m_{01}\|(0,0)\}$	$\{m_{21}\|(0,0)\}$
M_1	1	1	1
M_2	1	−1	−1
M_3	−1	−1	1
M_4	−1	1	−1

$\overline{\Gamma M}$

	$\{m_{01}\|(0,0)\}$
SM_1	1
SM_2	−1

$\overline{\Gamma K}$

	$\{m_{1\bar{1}}\|(0,0)\}$
LD_1	1
LD_2	−1

$\overline{KM}$

	$\{m_{21}\|(0,0)\}$
Y_1	1
Y_2	−1

TABLE S21: The relevant irreducible representations of $p6mm$ at the high-symmetry points $\Gamma = (0,0)$, $M = (1/2, 0)$, and $K = (1/3, 1/3)$, as well as along the lines $\overline{\Gamma M}$, $\overline{\Gamma K}$, and $\overline{KM}$. Note that $z = \exp(i\pi/3)$.

The fragile roots in $p6mm$ [S4] are given in Tab. S22; the elementary band representations can be retrieved from the Bilbao server.

#	root	# of bands	type
1	$\Gamma_1+\Gamma_2+K_1+K_2+M_3+M_4$	2	mirrors
2	$\Gamma_3+\Gamma_4+K_1+K_2+M_1+M_2$	2	mirrors
3	$\Gamma_3+\Gamma_4+K_3+M_1+M_2$	2	2D irrep & mirrors
4	$\Gamma_3+\Gamma_4+K_3+M_3+M_4$	2	2D irrep & mirrors
5	$\Gamma_1+\Gamma_2+K_3+M_3+M_4$	2	2D irrep & mirrors
6	$\Gamma_1+\Gamma_2+K_3+M_1+M_2$	2	2D irrep & mirrors
7	$\Gamma_6+K_1+K_2+M_1+M_2$	2	2D irrep & mirrors
8	$\Gamma_5+K_1+K_2+M_1+M_2$	2	2D irrep & mirrors
9	$\Gamma_5+K_3+M_3+M_4$	2	2D irrep (twice) & mirrors
10	$\Gamma_6+K_3+M_1+M_2$	2	2D irrep (twice) & mirrors
11	$\Gamma_6+K_1+K_2+M_3+M_4$	2	2D irrep & mirrors
12	$\Gamma_5+K_1+K_2+M_3+M_4$	2	2D irrep & mirrors

TABLE S22: Fragile roots and their types in wallpaper group $p6mm$.

2. *Bundling strategies*

Let us start with the first two roots #1 and #2, where with $\Gamma_{1/2}$, $K_{1/2}$, and $M_{1/2/3/4}$ only one dimensional irreps with real characters are involved. It is easy to check, that the mirrors along the high symmetry lines force us to bundle root #1 as

$$(\Gamma_1+K_1+M_3)\oplus(\Gamma_2+K_2+M_4). \tag{S56}$$

We now invoke the usual Chern argument by writing [S6]

$$e^{\mathrm{i}\pi C/3}=\prod_{i\,\in\,\mathrm{occ.}}(-1)^F\eta_i(\Gamma)\vartheta_i(K)\zeta_i(M), \tag{S57}$$

where $F=2S$, with S the total spin of the particles, η, ϑ, and ζ are the eigenvalues of $\{6^+|(0,0)\}$ and $\{3^+|(0,0)\}$, and $\{2|(0,0)\}$ respectively. In the following, we set $F=0$. For the bands above, this means, the Chern number C of these bands is given by

$$C=3+6n \quad \text{with} \quad n\in\mathbb{Z}. \tag{S58}$$

The only way this is compatible with time reversal symmetry is by having a band touching between the sets of bands. The same holds for root #2.

For roots #3–#6, the two-dimensional irrep at K glues the bands together into a bundle of two. Moreover, the mirrors along the lines to M and Γ fix the connectivity. For the roots #7, #8, #11, #12, the 2D irreps at Γ do the job, and again, the mirrors to M and K determine the connectivity to their irreps. For the roots #9 and #10, the bands are degenerate both at Γ and K.

Finally, the types of the roots of $p6mm$ are indicated in the last column of Tab. S22.

3. *Examples*

[S1] J. D. Joannopoulos, S. G. Johnson, J. N. Winn, and R. D. Meade, *Photonic crystals, molding the flow of light* (Princeton University Press, 2008).

[S2] K. K. Gomes, W. Mar, W. Ko, F. Guinea, and C. H. Manoharan, *Designer Dirac fermions and topological phases in molecular graphene*, Nature **483**, 306 (2012).

[S3] M. Greiner, O. Mandel, T. Esslinger, T. W. Hänsch, and I. Bloch, *Quantum phase transition from a superfluid to a Mott insulator in a gas of ultracold atoms*, Nature **415**, 39 (2002).

[S4] Z.-D. Song, L. Elcoro, and B. A. Bernevig, *Twisted bulk-boundary correspondence of fragile topology*, Science **367**, 794 (2020).

[S5] L. Fu and C. L. Kane, *Time reversal polarization and a Z_2 adiabatic spin pump*, Phys. Rev. B **74**, 195312 (2006).

[S6] C. Fang, M. J. Gilbert, and B. A. Bernevig, *Bulk topological invariants in noninteracting point group symmetric insulators*, Phys. Rev. B **86**, 115112 (2012).

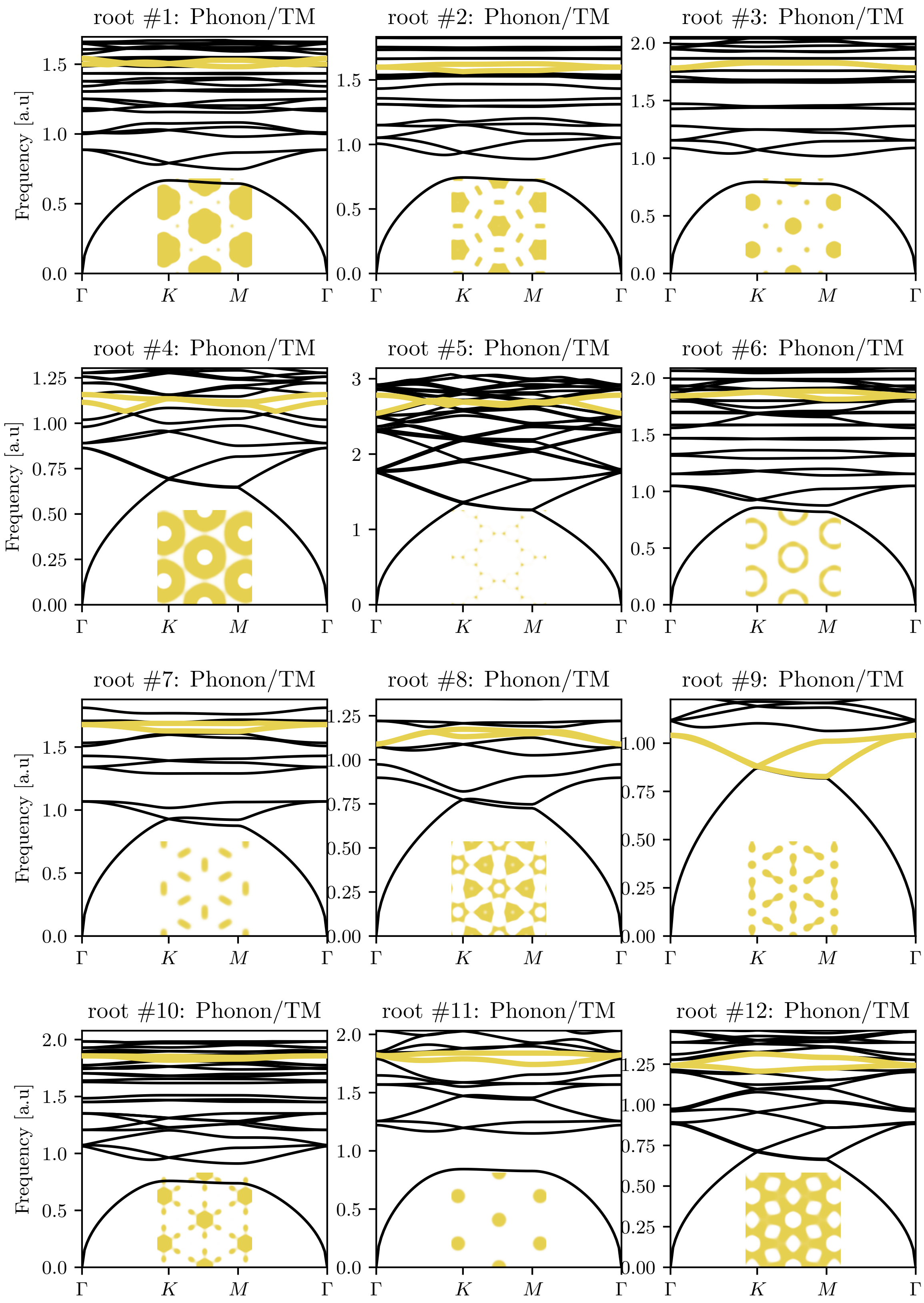

FIG. S42: One sample for each of the roots in $p6mm$ for phonons and TM photons.

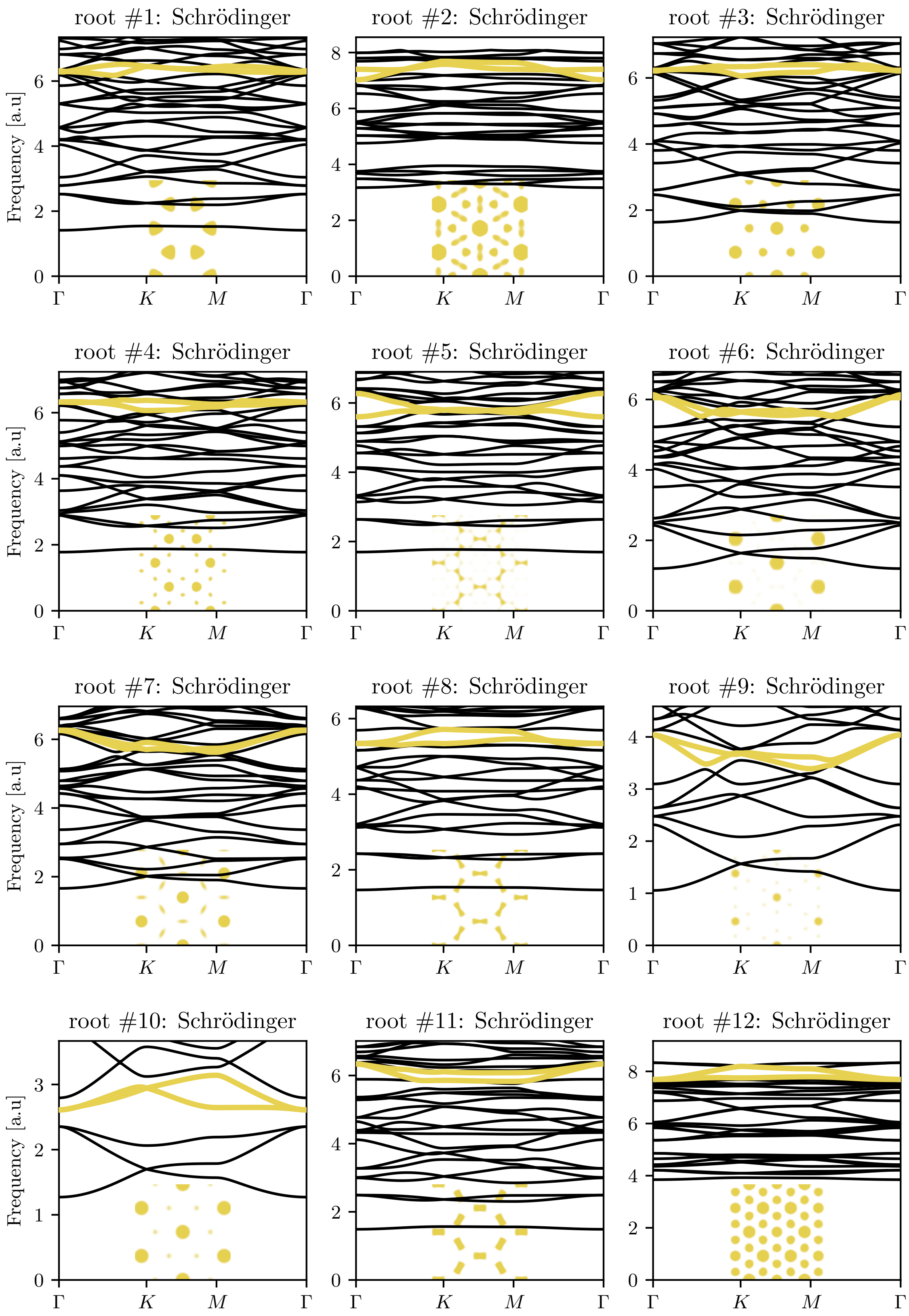

FIG. S43: One sample for each of the roots in $p6mm$ for systems described by the Schrödinger equation.

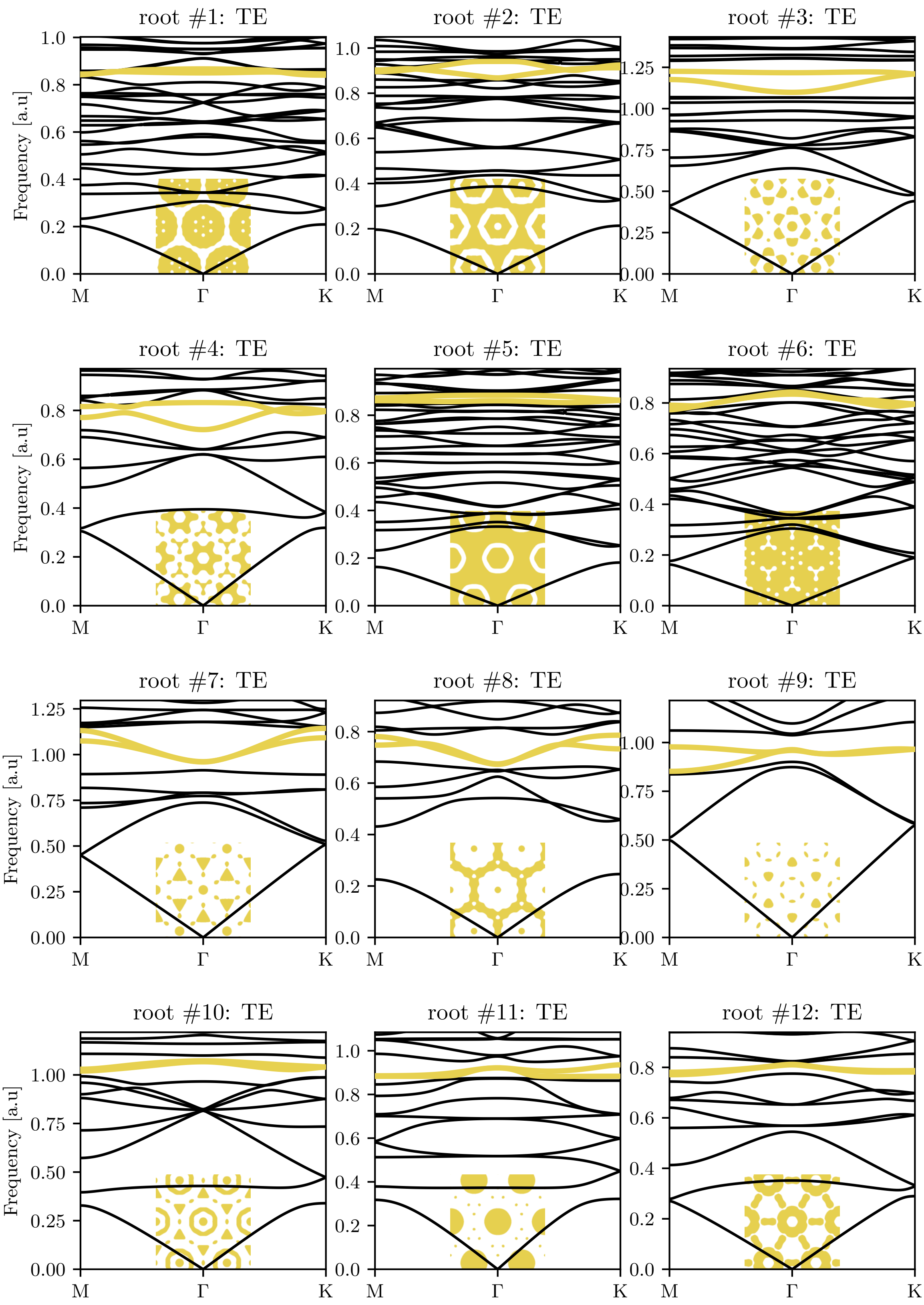

FIG. S44: One sample for each of the roots in $p6mm$ for TE photons.
